# TASOR is a pseudo-PARP that directs HUSH complex assembly and epigenetic transposon control

**DOI:** 10.1038/s41467-020-18761-6

**Published:** 2020-10-02

**Authors:** Christopher H. Douse, Iva A. Tchasovnikarova, Richard T. Timms, Anna V. Protasio, Marta Seczynska, Daniil M. Prigozhin, Anna Albecka, Jane Wagstaff, James C. Williamson, Stefan M. V. Freund, Paul J. Lehner, Yorgo Modis

**Affiliations:** 1 Molecular Immunity Unit, Department of Medicine, University of Cambridge, MRC Laboratory of Molecular Biology, Cambridge Biomedical Campus, Cambridge, CB2 0QH UK; 2 Cambridge Institute of Therapeutic Immunology & Infectious Disease (CITIID), University of Cambridge School of Clinical Medicine, Cambridge, CB2 0AW UK; 3 Structural Studies Division, MRC Laboratory of Molecular Biology, Cambridge Biomedical Campus, Cambridge, CB2 0QH UK; 4 Present Address: Department of Experimental Medical Science, Lund University, Lund, Sweden; 5 Present Address: The Gurdon Institute, Cambridge, UK; 6 Present Address: Department of Pathology, University of Cambridge, Cambridge, UK; 7 Present Address: Molecular Biophysics and Integrated Bioimaging Division, Lawrence Berkeley National Laboratory, Berkeley, CA 94720 USA; 8 Present Address: MRC Laboratory of Molecular Biology, Cambridge, UK

**Keywords:** Chromatin immunoprecipitation, Histone post-translational modifications, Gene silencing, Transcriptional regulatory elements, X-ray crystallography

## Abstract

The HUSH complex represses retroviruses, transposons and genes to maintain the integrity of vertebrate genomes. HUSH regulates deposition of the epigenetic mark H3K9me3, but how its three core subunits — TASOR, MPP8 and Periphilin — contribute to assembly and targeting of the complex remains unknown. Here, we define the biochemical basis of HUSH assembly and find that its modular architecture resembles the yeast RNA-induced transcriptional silencing complex. TASOR, the central HUSH subunit, associates with RNA processing components. TASOR is required for H3K9me3 deposition over LINE-1 repeats and repetitive exons in transcribed genes. In the context of previous studies, this suggests that an RNA intermediate is important for HUSH activity. We dissect the TASOR and MPP8 domains necessary for transgene repression. Structure-function analyses reveal TASOR bears a catalytically-inactive PARP domain necessary for targeted H3K9me3 deposition. We conclude that TASOR is a multifunctional pseudo-PARP that directs HUSH assembly and epigenetic regulation of repetitive genomic targets.

## Abstract (web summary)

The HUSH complex plays a key role in controlling transcription of viruses and transposable elements. Here, the authors define the biochemical basis of HUSH assembly and show that the TASOR subunit contains a pseudo-PARP domain critical for HUSH-dependent transgene repression and H3K9me3 deposition over targets genome wide.

## Introduction

Post-translational modification of histones and other chromatin proteins is a central mechanism by which eukaryotic cells regulate chromatin architecture and tune the dynamics of DNA-templated processes. One conserved example is trimethylation of histone H3 lysine 9 (H3K9me3), an epigenetic mark typically associated with low levels of transcription^1^. H3K9me3 marks repetitive regions of eukaryotic chromosomes where it presents a binding site for heterochromatin protein 1 (HP1)^2^. HP1 undergoes liquid-liquid phase separation to form a chromatin compartment that somehow excludes RNA polymerase from the DNA^3^. However, H3K9me3 is also present in transcriptionally-active euchromatin^4 ^— for example, over the bodies of certain protein-coding genes^5^. The mark is, therefore, central in maintaining genome stability and controlling transcriptional programs. In mammals, its importance at an organismal level is underlined by recent observations that dynamic regulation of H3K9me3 — catalyzed by multiple lysine *N*-methyltransferases — is critical for murine development^6^.

Genetic experiments studying position-effect variegation (PEV) in model organisms have identified much of the machinery involved in the formation of H3K9me3 domains^7^. Such position effects refer to the influence of local chromatin environment on gene expression. Forward genetic screens for mutations disrupting PEV in *Drosophila* revealed conserved factors required for heterochromatin formation, including HP1 itself^8^. Analogously, a mutagenic screen in a mouse line with a transgene reporter displaying variegated expression identified numerous epigenetic regulators, the ‘Modifiers of murine metastable epialleles’ (*Mommes*), several of which are specific to mammals^9,10^. A forward genetic screen that we conducted previously with an integrating lentiviral reporter identified the human silencing hub (HUSH) as a novel regulator of PEV in human cells^11^. HUSH is a complex of three proteins: transgene activation suppressor (TASOR), M-phase phosphoprotein 8 (MPP8), and Periphilin (PPHLN1, isoform 2). The activity of TASOR is critical in early development: the homozygous mutation L130P, at a conserved leucine in mouse TASOR (identified as *MommeD6*), is lethal in embryos before the completion of gastrulation^12^. The HUSH complex recruits the H3K9 methyltransferase SET domain bifurcated 1 (SETDB1) to deposit H3K9me3 (ref. ^11^) and the ATPase MORC2 to compact chromatin^13,14^. HUSH is a vertebrate-specific chromatin regulator that represses both exogenous and endogenous genetic elements. As well as targeting integrating lentiviruses, HUSH targets full-length transcriptionally-active retrotransposons including LINE-1s^15,16^, and cell-type specific genes such as zinc finger transcription factors (*ZNF*s)^11,14,15^. HUSH is also recruited, via the DNA-binding protein NP220, to repress expression of unintegrated murine leukemia virus^17^. The critical role of HUSH in antiretroviral immunity is highlighted by findings that primate lentiviral accessory proteins Vpr and Vpx target HUSH complex proteins for proteasome-mediated degradation^18–20^.

In the current model of HUSH-mediated repression, HUSH regulates both reading and writing of H3K9me3 (ref. ^14^). The MPP8 chromodomain binds K9-trimethylated H3 tail peptide^21^ and H3-like mimic sequences found in other proteins, including ATF7IP, the nuclear chaperone of SETDB1 (ref. ^22^). While these data suggest that MPP8 binding to methylated ATF7IP recruits SETDB1 to spread H3K9me3 over HUSH targets^23^, a read-write mechanism for H3K9me3 spreading involving MPP8 and ATF7IP/SETDB1 must be too simplistic: TASOR and Periphilin are both essential for HUSH-dependent lentiviral reporter repression, and the MPP8 chromodomain is required for establishment but not maintenance of repression^11^. Furthermore, HUSH targets are enriched within transcriptionally-active chromatin^15,16^, in contrast to classical heterochromatin regulators. Hence, key unanswered questions are: how TASOR and Periphilin contribute to HUSH targeting and repression; how H3K9 methylation by SETDB1 is regulated; and whether there are distinct mechanisms to recruit SETDB1 methyltransferase activity to HUSH loci, for example, at different stages of development.

Here we report multiscale biochemical and functional analyses of TASOR that provide new mechanistic insights into how HUSH assembles and regulates its targets. We report on the role of Periphilin elsewhere^24^. TASOR is a 1670-amino acid nuclear protein lacking functional annotations. At the molecular level TASOR remains poorly characterized, apart from its identification as an mRNA binding protein^25,26^. Here we show that TASOR is the central assembly platform of HUSH, providing binding sites for MPP8 and Periphilin. Targeted epigenomic profiling experiments support the model that TASOR binds and regulates H3K9me3, specifically over LINE-1 repeats and repetitive exons of transcribed genes. Analysis of HUSH domain organization reveals striking homology with the yeast RNA-induced transcriptional silencing (RITS) complex, and in a proteomic screen, we find TASOR associates with RNA processing components. Together with observations that transgene transcription enhances HUSH binding^15^, these data suggest that an RNA intermediate may be important for HUSH activity. Our cellular assays map the specific subdomains of TASOR and MPP8 necessary for HUSH assembly and transgene repression. Structural and biochemical studies reveal that TASOR contains a catalytically inactive poly-ADP ribose polymerase (PARP) domain that is dispensable for assembly and chromatin targeting but critical for epigenetic regulation of target elements. We find that this activity relies on an extended, dynamic loop that is unique in the PARP family. Our data demonstrate TASOR is a pseudo-PARP that governs both HUSH assembly and H3K9me3 deposition over repetitive genomic targets.

## Results

### TASOR regulates H3K9me3 over L1Ps and repetitive exons

We originally identified HUSH as a repressor of lentiviral transgenes^11,14^. Chromatin immunoprecipitation sequencing (ChIP-seq) showed that HUSH also targets endogenous genes and transposable elements^14–16^. Notably, transcription promotes target binding by the HUSH subunit MPP8 (ref. ^15^), and HUSH loci were found in transcriptionally-active euchromatic regions as defined by epigenetic marks^15,16^ and sensitivity to sonication^4^. These results highlight that H3K9me3 is not restricted to heterochromatin and suggest that sonication of cross-linked chromatin in ChIP protocols could influence analysis of HUSH regulation. TASOR ChIP-seq also displayed low sensitivity^15^. For these reasons, we applied orthogonal and targeted strategies for TASOR epigenomic profiling, CUT&RUN^27^ and CUT&Tag^28^.

**Fig. 1:**
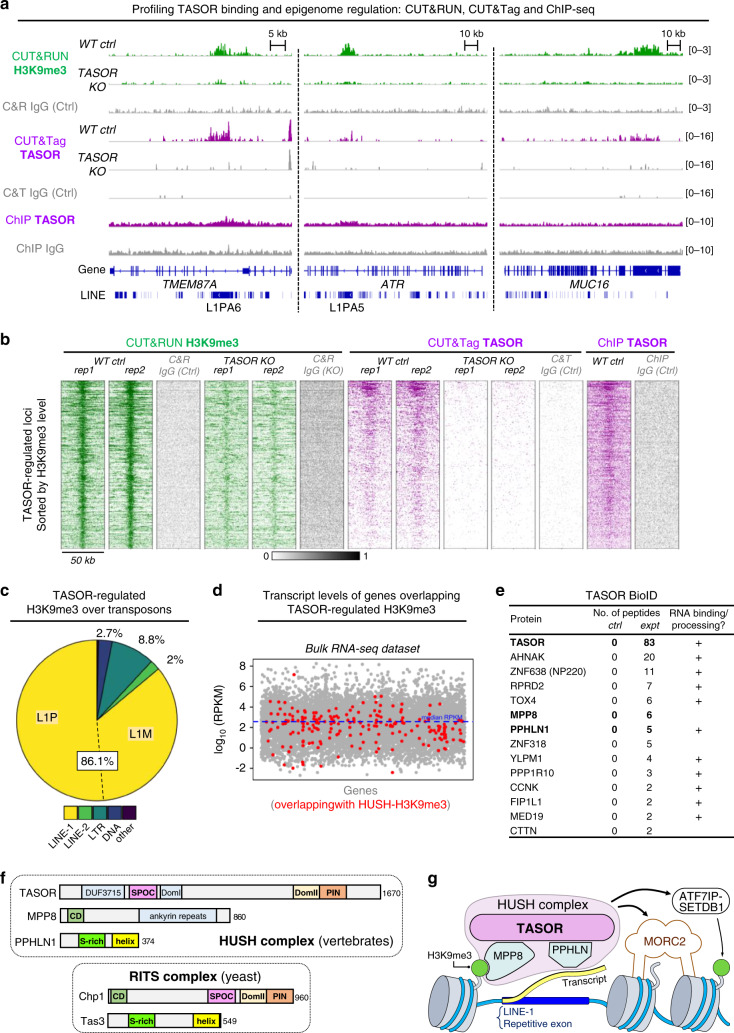
HUSH activities and domain structures resemble the RITS complex. **a** Genome browser snapshots of TASOR binding and regulation of H3K9me3. Shown are CUT&RUN H3K9me3 tracks in TASOR-positive and TASOR-negative HeLa cells (green); CUT&Tag and ChIP TASOR tracks in TASOR-positive (purple) and TASOR-negative cells; and an IgG control for each technique from control cells (gray). **b** Heatmaps of the signal from indicated experiments and replicates across 393 TASOR-regulated loci (rows). The sequencing depth of CUT&Tag TASOR experiments was ~4M reads, compared with ~55M reads for ChIP-seq. **c** Overlaps of 393 TASOR-regulated H3K9me3 peaks with different repeat classes. Since these peaks extend over several kilobases (mean length 6369 bp), several covered more than one annotated repeat. **d** Coding genes overlapping with TASOR-regulated H3K9me3 peaks were plotted (red dots) on a scatterplot showing gene transcript levels. Raw data were processed from triplicate RNA-seq data on wild-type HeLa cells, mapped to hg38 (ref. ^14^). RPKM, reads per kilobase per million mapped reads. **e** TASOR BioID hits. HUSH subunits are marked in bold. Control refers to TASOR knockout cells treated with biotin but not expressing BirA-tagged TASOR. **f** Predicted domain architecture of *Homo sapiens* HUSH subunits, showing similarity with *Schizosaccharomyces pombe* Chp1 and Tas3. DUF, domain of unknown function. SPOC, Spen ortholog C-terminal. PIN, PilT N-terminus. CD, chromodomain. HUSH, human silencing hub. RITS, RNA-induced transcriptional silencing complex. **g** Schematic model of HUSH repression.

CUT&RUN profiling of H3K9me3 in the presence and absence of TASOR identified 393 TASOR-regulated loci with high resolution and sensitivity (Fig. 1a, b and Supplementary Fig. 1a). The proportion of global H3K9me3 regulated by HUSH — approximately 1% — was comparable to that determined by ChIP-seq^11,15^. We observed a strong association with LINE-1 repeats (L1s) in our analysis. Of all the transposable element classes overlapped by TASOR-regulated sites, 86.1% corresponded to L1s of which the majority were primate-specific L1Ps (Fig. 1c). TASOR CUT&RUN was unsuccessful, perhaps due to TASOR’s size and poor solubility, but CUT&Tag gave comparable signal to ChIP-seq with an order of magnitude lower sequencing depth (Fig. 1a, b). We found that the strongest TASOR ChIP and CUT&Tag peaks were co-occupied by H3K9me3, consistent with the model that TASOR binds chromatin via MPP8 (Fig. 1b and Supplementary Fig. 1b). Secondary H3K9me3-independent association was also observed at a handful of sites by CUT&Tag (Supplementary Fig. 1c). The resolution afforded by targeted methods enabled boundaries of TASOR binding and associated H3K9me3 deposition to be mapped with precision, revealing that this often coincides with boundaries of L1P sequences (Fig. 1a and Supplementary Fig. 1b). Although most HUSH loci contain H3K9me3, other epigenetic marks likely contribute to HUSH complex genome association. Indeed, H3K9me3 contributes to multiple chromatin states, partly in combination with other histone modifications, which we do not investigate here. For example, local acetylation patterns may provide an additional selectivity filter for HUSH targeting^15^ and SETDB1 methyltransferase activity^29^.

Intronic L1Ps account for most but not all TASOR-regulated H3K9me3 peaks that overlap with genes. ZNF (zinc finger) genes are significantly enriched (*P* < 2.2 × 10^−16^, Fisher exact test), consistent with other data sets^11,15^. TASOR-regulated H3K9me3 over *ZNF*s predominantly covers their 3’ exons, which encode repetitive and rapidly-evolving zinc finger arrays^5,30^. Inspection of other non-L1 targets suggested that TASOR-dependent H3K9me3 deposition may be found over exons encoding repetitive polypeptides irrespective of gene organization (e.g., *MUC16* exon 3, *BRCA2* exon 11, *C2orf16* exon 1, *AHNAK* exon 5, *CELSR3* exon 1) (Fig. 1a and Supplementary Fig. 1d). Repetitive or rapidly-evolving genes are sources of recombination and genome damage^5,31^, a potential shared by L1Ps. Together, these observations suggest that HUSH-dependent H3K9me3 deposition and chromatin compaction could have a genome protective function alongside its established repressive function.

### TASOR binds transcribed genes and RNA processing machinery

Retroviral and LINE-1 HUSH targets pose a threat to the genome by replication through an RNA intermediate. Induced LINE-1 transcription promoted MPP8 genome binding in K562 cells^15^ and a subset of HUSH-bound genes were found to be regulated in a tissue-specific manner^15,16^. According to reanalysis of RNA-seq data^14^, the median expression of genes overlapping TASOR-regulated sites (RPKM = 7.52, *n* = 228) is comparable to that of all other genes (RPKM = 7.71, *n* = 14,211) (Fig. 1d). Closer examination supports an association between transcription and H3K9me3 deposition through HUSH. For example, *MUC16* is expressed in HeLa but not K562 cells^15^ and is only H3K9me3-marked in a TASOR-dependent manner in HeLa cells. *BRCA2* is expressed in HeLa and K562 and is HUSH-modified in both lines^15^ (Fig. 1a and Supplementary Fig. 1d). Our data support a model in which transcription of LINE-1s or other repetitive exons correlates with TASOR binding and H3K9me3 deposition over the element.

To investigate associations made by TASOR on chromatin, we performed proximity-dependent labeling (BioID) using BirA-tagged TASOR in TASOR knockout (KO) cells (Fig. 1e and Supplementary Data 1). Using this approach, we identified peptides from TASOR, MPP8, Periphilin and 10 other chromatin-associated proteins. Among our top hits were matrin-type zinc finger proteins ZNF318 and ZNF638 (NP220). Although NP220 is known to recruit HUSH to unintegrated murine retroviral DNA^17^, our data predict additional roles for this interaction in the absence of infection. We also detected RPRD2, a regulator of RNA Polymerase II previously identified alongside TASOR as a repressor of LINE-1s^15^ and HIV^32^. HUSH effectors MORC2 and the SETDB1/ATF7IP complex were absent from the list, suggesting that these factors interact with HUSH transiently and possibly indirectly (e.g., through chromatin). Also absent were proteins usually associated with transcriptionally-inert heterochromatin, reinforcing the model that HUSH resides at a subset of H3K9me3-marked sites. 11 of the 14 proteins we identified are annotated RNA-binding proteins. Several proteins identified (e.g., CCNK, MED19, RPRD2, FIP1L1, PPP1R10, and TOX4) have been associated with regulating mRNA processing or RNA polymerase II activity. Association with RNA processing machinery is consistent with observations that HUSH binds and regulates transcriptionally-active genomic targets, together with TASOR’s annotation as an mRNA-interacting protein^25,26^.

### HUSH resembles the yeast RITS complex

Despite its important roles in antiviral defense and vertebrate development, TASOR lacks functional annotations in its 1670-residue sequence apart from a ‘domain of unknown function’ (DUF3715, residues 106–332). Disorder^33^ and structural homology^34,35^ prediction on the primary sequence of TASOR identified four additional putative domains (Fig. 1f and Supplementary Fig. 2a, b). A Spen paralog and ortholog C-terminal (SPOC) beta barrel domain (residues 350–505) is predicted to lie adjacent to DUF3715, while no structural homology was identified for the third ordered region (referred to as DomI, residues 525–633). Residues 1233-1466 exhibit homology to the DomII and PIN domains of the *S. pombe* protein Chp1 (Supplementary Fig. 2a). Intriguingly, Chp1 also contains a SPOC domain (Supplementary Fig. 2b) and, like MPP8, an H3K9me3-binding chromodomain. Furthermore, the Chp1 binding partner Tas3 is a small protein that resembles Periphilin (Fig. 1f).

Together Chp1 and Tas3 form the core structure of the yeast RNA-induced transcriptional silencing (RITS) complex^36^. The striking resemblance between the domain organization of RITS (Chp1-Tas3) to that of HUSH (Fig. 1f) is particularly notable given the functional similarities between the two complexes. In yeast, Tas3 self-associates through C-terminal helical repeats to spread heterochromatic gene silencing^37^. Periphilin self-association — through disordered and C-terminal helical regions — is likewise required for HUSH repression in human cells^24^. RITS targets repetitive sequences in centromeres and telomeres, and — as predicted for HUSH (Fig. 1g) — mediates deposition of repressive H3K9me3 over repeat elements in response to transcription^36,38^.

### Mapping regions in TASOR and MPP8 required for HUSH activity

**Fig. 2:**
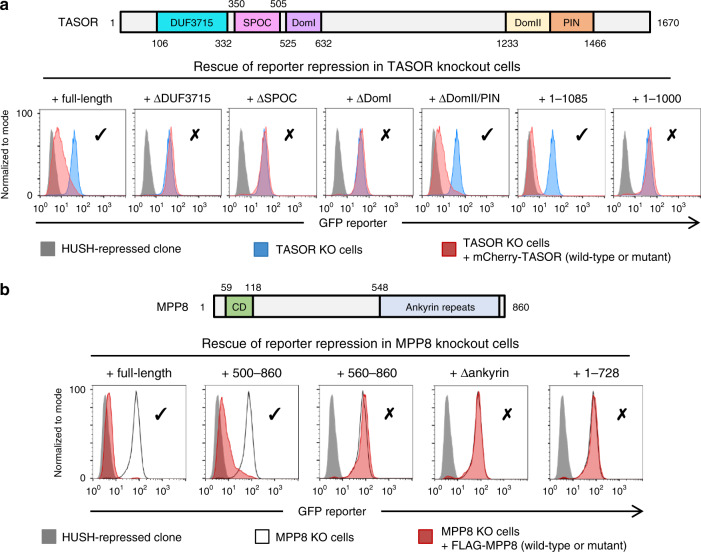
Functional requirements of TASOR and MPP8 domains in HUSH transgene repression. **a** Genetic complementation of TASOR knockout cells. Exogenous expression of full-length TASOR or TASOR lacking the DomII/PIN domain restores GFP transgene repression as measured by flow cytometry, but mutants lacking the DUF3715, SPOC or DomI domains are non-functional. TASOR(1–1085) is functional and TASOR(1–1000) is non-functional. **b** Genetic complementation assays in MPP8 knockout cells demonstrate that the C-terminal ankyrin repeats are required for function, but the first 499 residues are dispensable. Ticks and crosses indicate functionality in this assay.

Next we assessed which TASOR and MPP8 domains are required for HUSH transgene repression. We first generated a panel of TASOR truncation mutants (Supplementary Fig. 3a) and performed genetic complementation assays in TASOR knockout cells harboring a de-repressed GFP lentiviral transgene (Fig. 2a). Upon expression of full-length TASOR, HUSH function was restored and the reporter was repressed. However, TASOR deletion mutants lacking the DUF3715, SPOC, or DomI domains were non-functional. A variant lacking the DomII/PIN domains (deletion of residues 1233–1670) complemented the knockout, indicating that this C-terminal region is not required for transgene repression under the conditions tested. We note that DomII/PIN is nonetheless highly conserved in TASOR orthologues, and these domains may therefore play functional roles not captured by our assay. Indeed, the PIN domain of Chp1 was required for repression by RITS at subtelomeric but not centromeric repeats^36^. Finally, a variant with additional C-terminal truncation — TASOR(1–1085) — retained function, but TASOR(1–1000) did not.

The domain structure of MPP8 is comparatively simple: an N-terminal chromodomain separated from C-terminal ankyrin (helix-loop-helix) repeats by a linker. Surprisingly, we previously found the chromodomain to be dispensable for the maintenance of HUSH function, although a mutation that inhibits H3K9me3 binding delayed re-establishment of reporter repression^11^. Extending this analysis of MPP8, we found that the first 499 amino acids could be removed without further impairing HUSH function. However, deletion of an additional 60 residues or the C-terminal ankyrin repeats did abolish HUSH function (Fig. 2b and Supplementary Fig. 3b). We conclude that the DUF3715, SPOC, and DomI domains of TASOR, along with its central linker, are required to maintain transgene repression by HUSH, but the DomII/PIN and C-terminus are dispensable. The C-terminal portion (500–860) of MPP8, which contains the predicted ankyrin repeats, is likewise required.

### TASOR lies at the heart of the HUSH complex

**Fig. 3:**
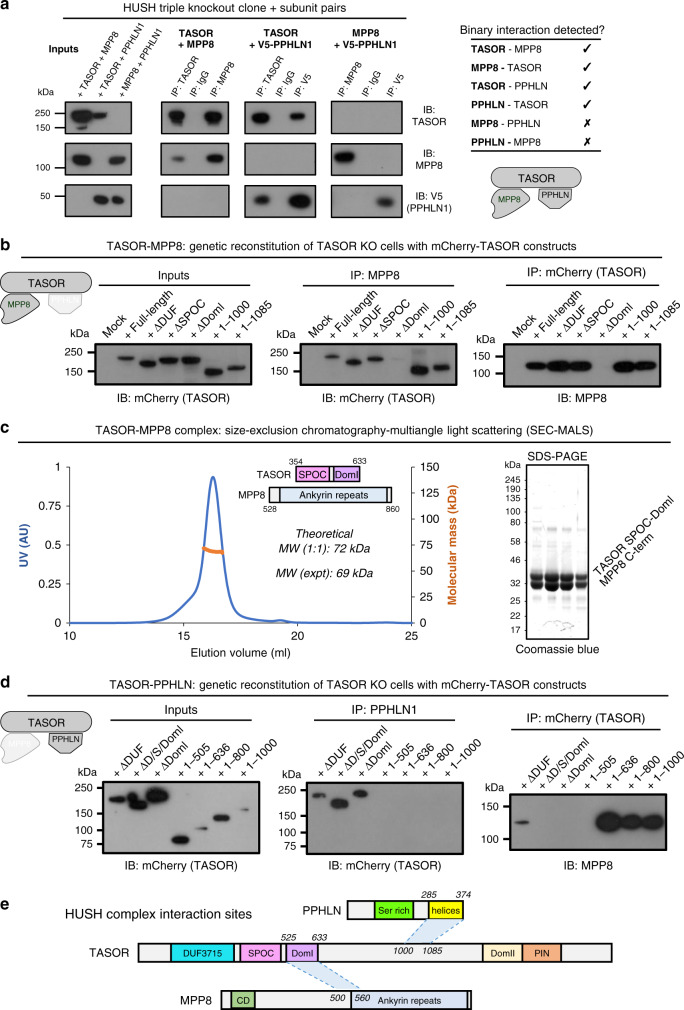
TASOR lies at the heart of the HUSH complex. **a** TASOR interacts separately with MPP8 and Periphilin to mediate assembly of the HUSH complex. Pairwise combinations of HUSH subunits were exogenously expressed in HUSH triple KO cells, and interactions detected by co-immunoprecipitation (co-IP) followed by immunoblot. **b** TASOR DomI mediates the interaction with MPP8. The indicated TASOR truncation mutants were exogenously expressed in TASOR KO cells and their association with endogenous MPP8 assessed by co-IP. **c** SEC-MALS of recombinant TASOR(354–633)-MPP8(528–860) complex (theoretical MW, 72 kDa for a 1:1 complex) with representative SDS-PAGE gel of SEC peak fractions (inset). **d** The central linker of TASOR mediates the interaction with Periphilin. **e** Summary of core HUSH interaction sites.

TASOR domains essential for HUSH function could (i) mediate HUSH complex formation through interactions with MPP8 and Periphilin or (ii) have biological activities necessary for repression (or recruit effector proteins with these activities). We first considered the overall role of TASOR in HUSH assembly. Starting from a cell line lacking all three HUSH subunits^11^, we re-expressed subunits in pairwise combinations and examined their interactions through reciprocal co-immunoprecipitation (co-IP) (Fig. 3a). MPP8 and Periphilin precipitated with TASOR but no binding was detected between MPP8 and Periphilin without TASOR. We conclude that TASOR lies at the heart of the core HUSH complex.

To map binding sites, we performed co-IP of endogenous MPP8 with tagged TASOR truncation variants or vice versa. TASOR DomI was required for MPP8 binding regardless of which protein was immunoprecipitated (Fig. 3b). The adjoining SPOC domain was dispensable for MPP8 binding in cells, which was unexpected since SPOC domains from other transcriptional regulators like yeast Chp1 form stabilizing protein–protein interactions through an exposed hydrophobic patch^36,39^. Attempts to isolate recombinant TASOR SPOC or DomI domains, or DomI-MPP8 complexes, resulted in poor yields of soluble protein. However, we were able to purify a stable, 1:1 TASOR-MPP8 complex following recombinant co-expression of a tandem TASOR SPOC-DomI construct (residues 354–633) and the MPP8 C-terminus (residues 528–860) (Fig. 3c). Together these data support a binding mode in which the TASOR SPOC domain stabilizes DomI and that the interaction with MPP8 occurs through a hydrophobic interface. Since MPP8(500–860) is functional but MPP8(560-860) is non-functional (Fig. 2b), our data suggest that the minimal TASOR binding site lies between MPP8 residues 500–560.

To assess the TASOR–Periphilin interaction, we immunoprecipitated endogenous Periphilin and blotted for mCherry-tagged TASOR constructs. Periphilin pulled down TASOR mutants lacking the DUF3715, SPOC, and DomI domains, but not TASOR(1–1000) or TASOR variants with longer C-terminal truncations (Fig. 3d). We noted some variability in the abundance of TASOR variants. TASOR(1-636) and TASOR(1–1000) were barely detectable suggesting these variants are unstable. There was no correlation between the relative expression level of the variants and their ability to support HUSH complex assembly. Given that TASOR(1–1085) is functional, these data suggest that the Periphilin binding site lies in residues 1000–1085. Indeed we report elsewhere the crystal structure of a minimal TASOR–Periphilin complex, showing that TASOR(1014–1095) contributes directly to binding the Periphilin C-terminus^24^. Together these results delineate the biochemical requirements for HUSH assembly, and suggest that the central portion of TASOR, spanning residues 350–1085, is a sufficient assembly scaffold (Fig. 3e).

### TASOR DUF3715 is a PARP domain

Having established that TASOR acts as the core member of HUSH and defined the domains necessary for assembly, TASOR’s N-terminal DUF3715 stood out as significant for several reasons: (i) it is required for transgene repression in a manner independent of assembly; (ii) it is the domain that differentiates TASOR from *S. pombe* Chp1 and (iii) it contains the embryonic lethal mouse mutation (L130P)—underlining its key functional role at an organismal level. Bioinformatic analysis suggested that DUF3715 resembles a poly ADP-ribose polymerase (PARP) catalytic domain. Functions of the PARP family, which contains at least 18 members in humans, include response to genome damage and viral infection^40^. This is notable given TASOR’s role as a LINE-1 and viral repressor.

**Fig. 4:**
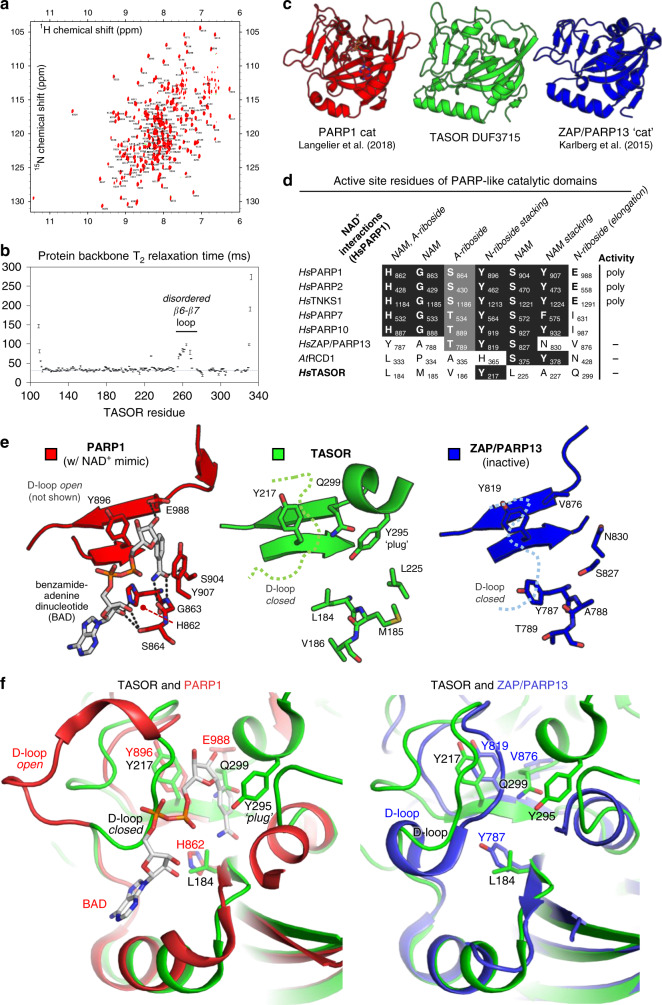
TASOR DUF3715 is a PARP domain. **a** Assigned ^1^H-^15^N BEST TROSY spectrum of ^15^N-labeled TASOR(106–332) at 293 K. **b** Backbone T_2_ relaxation times of TASOR(106–332). The disordered β6–β7 loop that was removed to promote crystallization is marked. Source data are provided as a Source data file (“T2 relaxation data” tab). **c** Overall structures of PARP domains of human PARP1 (red), TASOR (green), and ZAP (blue). **d** Alignment of PARP active sites and NAD^+^ binding residues for a selection of PARP family members. NAM, nicotinamide; A-riboside, adenosyl-riboside; N-riboside, nicotinamide-riboside. **e**, **f** Comparison of active sites of the structures shown in (**c**), with key features marked.

We, therefore, aimed to study the structure and biochemical properties of DUF3715. Despite extensive trials, we could not induce the wild-type DUF3715 to crystallize. We used NMR spectroscopy to gain insight into its structural and dynamic properties and recorded well-resolved ^1^H, ^15^N correlation spectra on ^15^N-labeled protein (Fig. 4a). To obtain peak assignments we required sidechain deuteration and expressed the domain in media prepared with deuterated water (^2^H_2_O). Upon purification in (^1^H) aqueous solvents, several peaks in the spectrum were missing, consistent with a rigid core in which the rate of amide H-D exchange is exceptionally slow (>5 days). A partial denaturation-refolding protocol enforced exchange of core amide deuterons (Supplementary Fig. 4a), enabling assignment of 184 (of 221) non-proline backbone amide resonances. Secondary structure prediction from chemical shifts^41^ confirmed an α/β PARP fold with loop insertions (Supplementary Fig. 4b).

### Overall structure and dynamics of the TASOR PARP domain

NMR assignments allowed us to measure T_1_ and T_2_ relaxation times to investigate domain dynamics (Fig. 4b). The average T_1_/T_2_ ratio is proportional to overall tumbling correlation time τ_C_, which we determined to be ~15 ns. This value suggests DUF3715 is largely monomeric in solution at 250 µM, though the oligomeric status of full-length TASOR remains unknown. Elevated T_2_ relaxation times for residues 255–270 (the loop connecting strands β6–β7) were indicative of motions faster than overall tumbling (τ_C_), in the ns-ps timescale^42 ^— commonly described as disorder (Fig. 4b). A TASOR variant with part of this disordered loop deleted (Δ261–269) retained transgene repression activity (Supplementary Fig. 4c).

Hypothesizing that β6–β7 loop disorder precluded crystallization of the wild-type domain, we purified a variant with the Δ261–269 deletion. Validating our design, crystals were readily grown of native and SeMet-labeled samples (Supplementary Fig. 4d), enabling X-ray structure determination of TASOR DUF3715 to a resolution of 2.0 Å by single-wavelength anomalous dispersion (SAD) phasing (Supplementary Table 1, Supplementary Fig. 4e). Comparison with published structures using the Dali server^43^ confirmed similarity of the overall fold with PARPs from various subclasses (Fig. 4c) including canonical poly- (*Hs*PARP1, PDB:6BHV), mono- (*Hs*PARP10, PDB:3HKV) and catalytically-inactive (*Hs*ZAP/PARP13, PDB:2X5Y) ADP-ribosyl transferases, and the inactive plant PARP homolog RCD1 (*At*RCD1, PDB:5NGO).

### The TASOR PARP domain lacks an NAD^+^ binding site

PARPs use NAD^+^ as a cofactor to catalyze addition of one or more ADP-ribosyl units onto target proteins, although some PARPs have lost this activity. Since mechanistic studies of ADP-ribosylation are hampered by a lack of understanding of PARP substrates, crystal structures have been a useful means to classify the family into active and inactive members^44^. The structure of PARP1 catalytic domain in complex with non-hydrolysable NAD^+^ analog benzamide adenosine dinucleotide (BAD) provides a near-complete picture of interactions made during NAD^+^ binding^45^. We tabulated amino acids making ligand contacts in the PARP1-BAD structure, including the canonical histidine-tyrosine-glutamate triad required for poly-ADP ribosylation (Fig. 4d, e). Substitution of the glutamate, as in PARP7 and PARP10, removes capacity for chain elongation, thus limiting these enzymes to mono-ADP-ribosylation^46^. The other residues involved in NAD^+^ binding are conserved in active PARPs, regardless of whether activity is mono- or poly-ADP ribosylation. By contrast, the human zinc finger antiviral protein (*Hs*ZAP, or PARP13) and *Arabidopsis* RCD1 lack two or more of the NAD^+^-binding residues and are catalytically inactive^47,48^. In TASOR the key residues required for catalysis are even more degenerated than in ZAP or RCD1: all but one of them are replaced by hydrophobic amino acids (Fig. 4d, f). TASOR shares a further similarity with ZAP/PARP13, in that the equivalent of its active site loop or D-loop (TASOR residues 200-210) adopts a closed conformation relative to PARP1, in which it is open^45,47^ (Fig. 4e, f). Together with a short helix spanning residues 294–298, these features occlude the TASOR ligand-binding pocket and the sidechain of Tyr295 plugs the nicotinamide binding cleft. Based on our structure, TASOR therefore lacks the chemical functionality and physical space to bind the NAD^+^ cofactor.

### TASOR is a pseudo-PARP that binds weakly to ssRNA

**Fig. 5:**
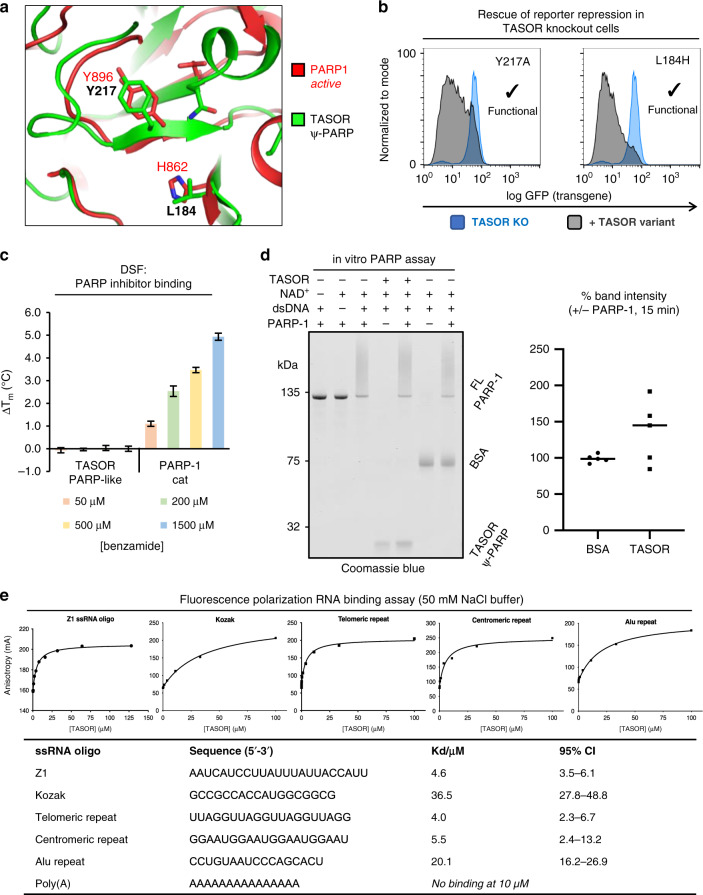
TASOR is a pseudo-PARP. **a** Active site comparison of PARP1 and TASOR, showing key functional residues. **b** Transgene repression assays show that mutation of the degenerated TASOR PARP active site does not affect TASOR function. **c** TASOR PARP domain does not bind promiscuous PARP inhibitor benzamide. DSF of three replicates, shown are mean ± standard deviation. **d** Full-length PARP1 auto-PARylates in the presence of dsDNA and NAD^+^ but TASOR PARP domain is not significantly affected. **e** Binding of 5’(6-FAM)-labeled ssRNA oligonucleotides to TASOR(106–332) by fluorescence polarization (FP), with a table summarizing the binding data. Source data for (**c**–**e**) are provided in the Source data file.

Consistent with our structural data illustrating a degenerate active site, point mutations removing the only remaining conserved amino acid (Y217A) or restoring a key NAD^+^ binding residue (L184H) did not affect TASOR-dependent transgene repression in cells (Fig. 5a, b). Differential scanning fluorimetry (DSF) experiments showed that while the domain was stable in isolation (T_m_ 51.5 °C), no change in T_m_ was observed upon addition of up to 1.5 mM benzamide, a promiscuous NAD^+^-mimic and PARP inhibitor (Fig. 5c). By contrast, the catalytic domain of PARP1 showed robust concentration-dependent stabilization as expected^45^. We then performed a gel-based poly-ADP-ribosylation (PARylation) assay by mixing recombinant full-length human PARP-1 with the TASOR PARP domain or negative control BSA. While PARP-1 robustly auto-PARylated in the presence of NAD^+^ and dsDNA, shown by a high molecular weight smear in the Coomassie-stained gel, we did not detect significant evidence of TASOR modification or auto-modification under the conditions tested (Fig. 5d). We conclude that TASOR’s active site is non-catalytic in isolation, like the one in human ZAP/PARP13 (ref. ^47^).

ZAP — another non-catalytic human PARP — functions by binding and degrading target viral RNAs^49^. Because of this and the association between TASOR and transcription (Fig. 1), we were interested to note that recombinant TASOR(106–332) coeluted with RNA from the *E. coli* expression host: bound nucleic acids were sensitive to treatment with high salt and RNase but not DNase (Supplementary Fig. 4f). The purified domain bound to various short ssRNA ligands with micromolar affinity in the presence of 50 mM NaCl, with limited sequence specificity (Fig. 5e). This interaction was further weakened at higher salt concentrations, suggesting that binding is non-specific and driven primarily by electrostatics. Nonetheless, it is plausible that the PARP domain contributes to RNA binding by full-length TASOR, or that the TASOR PARP domain binds an as yet unidentified RNA sequence with high affinity. RNA binding is consistent with the annotation of TASOR (along with Periphilin) as part of the HeLa mRNA interactome^25,26^, and association with mRNA processing machinery (Fig. 1e). It may be that as in ZAP, other domains in HUSH confer greater affinity or specificity to mRNA binding. Taken together, our structural and biochemical data show that TASOR contains a catalytically-inactive PARP domain and that, like ZAP and RCD1, TASOR may be considered a pseudo-PARP^50^.

### PARP loop with concerted motions required for HUSH functions

**Fig. 6:**
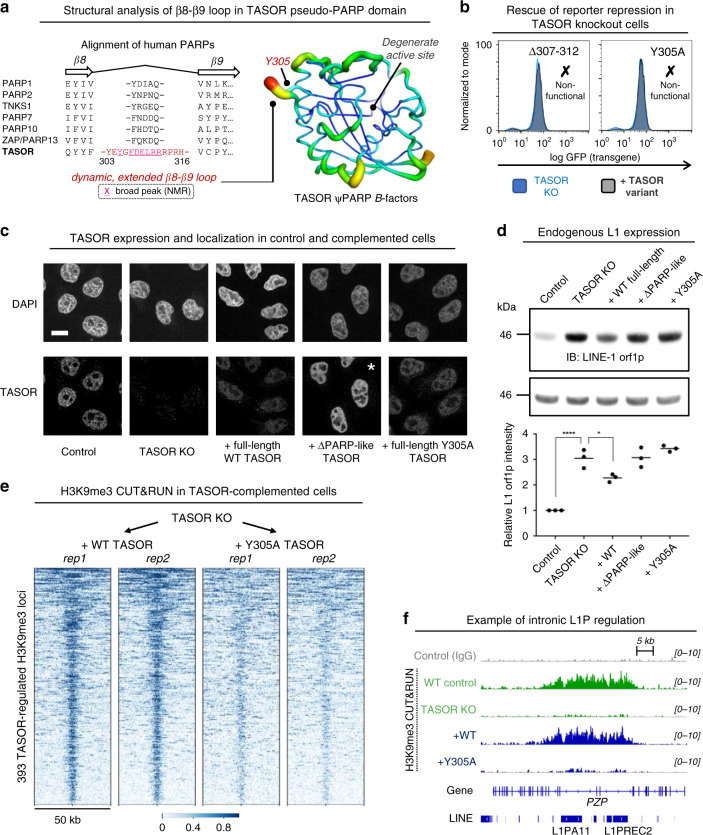
Structure-guided TASOR mutation abrogates HUSH repression, L1 restriction and H3K9me3 deposition. **a** Alignment of human PARP catalytic domains, focused on the connecting loop between the final two beta-strands. TASOR residues whose signals are broadened by fast T2 relaxation in NMR spectra are underlined. (left). Structure of TASOR pseudo-PARP domain colored by B-factor with features marked (right). **b** Δ307–312 or Y305A mutations in the β8–β9 extended loop of TASOR cause loss of GFP transgene repression. **c** Confocal immunofluorescence of TASOR localization in nuclei of indicated cell lines. Scale bar, 15 µm. Asterisk is shown for TASOR in ΔPARP cells; the expression was higher for this construct so brightness was adjusted to avoid saturation. **d** TASOR KO causes derepression of LINE-1 orf1 expression. Repression is partly rescued by full-length WT TASOR but not TASOR ΔPARP nor Y305A. *P < 0.05; ****P < 10^−4^ that KO and control or complemented cell lines are the same (*n* = 3, one-way ANOVA, measurements taken from distinct samples). Source data are provided in the Source data file (“L1 Westerns” tab). **e** CUT&RUN heatmaps showing H3K9me3 levels in TASOR KO cells complemented with either wild-type or Y305A TASOR. The Y305A mutation causes loss of H3K9me3 at the 393 TASOR-regulated loci identified in Fig. 1. **f** Genome browser snapshot of CUT&RUN data showing rescue of H3K9me3 over two intronic L1P elements in *PZP* in the presence of WT but not Y305A TASOR.

Topological differences between our structure and the canonical PARP fold are most pronounced in loops. In particular, the loop connecting the final two strands (β8 and β9 in TASOR) spans 14 residues in TASOR (Tyr303–His316), compared to 5 residues in all annotated human PARP family members (Fig. 6a). In TASOR the extended β8–β9 loop has elevated *B*-factors in the crystal structure, and broad NMR peaks caused by fast T_2_ relaxation (Figs. 4b, 6a). The latter is suggestive of conformational changes occurring on timescales slower than tumbling time τ_C_ (i.e., ms to µs). Such concerted motions require a higher activation energy and often correlate with functionally-relevant processes like conformational exchange^42^. This holds true for TASOR: variants Δ307–312 and Y305A were non-functional in our transgene repressor assay (Fig. 6b).

We then aimed to further dissect the functional consequences of the Y305A mutation. First, we purified the Y305A mutant to confirm that it did not cause domain misfolding (Supplementary Fig. 5a). DSF showed that the mutant (T_m_ 48.0 °C) was modestly destabilized compared to WT (T_m_ 51.5 °C) but fully-folded at physiological temperature. In cells, we confirmed that the Y305A mutant remained localized to the nucleus (Fig. 6c). Moreover, the ΔPARP deletion mutant remained chromatin-associated (Supplementary Fig. 5b) and both TASOR Y305A and ΔPARP also associated with the same set of cellular proteins as the WT protein in BioID experiments (Supplementary Fig. 5c). Together, these data are consistent with a model in which the pseudo-PARP domain is not required for chromatin localization nor HUSH assembly, although it remains possible that it contributes to TASOR targeting under certain conditions. Finally, we asked whether the Y305A mutation affected LINE-1 repression and TASOR-regulated H3K9me3 deposition. Expression of LINE-1 orf1p in TASOR KO cells was partially re-repressed by expression of WT TASOR but not the ΔPARP or Y305A variants (Fig. 6d). CUT&RUN profiling showed that the Y305A point mutation caused a near-complete loss of H3K9me3 deposition at the 393 sites we defined to represent TASOR-regulated H3K9me3, an effect functionally equivalent to TASOR KO (Fig. 6e, f). We conclude that the extended, dynamic β8–β9 loop — conserved in TASOR but unique among the human PARP family — is necessary not only for reporter transgene repression but also for genome-wide H3K9me3 deposition and LINE-1 repression by HUSH.

## Discussion

This study provides a molecular characterization of TASOR and how it contributes to HUSH function. We have shown that TASOR is as an assembly platform for MPP8 and Periphilin, and we have identified the minimal molecular determinants of HUSH complex assembly. TASOR(525–633) interacts with MPP8(500–560), and TASOR(1000–1085) interacts with Periphilin(285–374)^24^ (Fig. 3).

We report striking homology in the domain organization of the HUSH complex and the yeast RNA-induced transcriptional silencing (RITS) complex (Fig. 1). The core subunits of each complex, TASOR and Chp1, both contain a SPOC domain and C-terminal DomII/PIN domains. Both complexes also contain chromodomains — in MPP8 and Chp1, respectively — that recognize H3K9me3-marked chromatin. Chp1 binding partner Tas3 also resembles HUSH subunit Periphilin: both are largely unstructured with low-complexity sequences and C-terminal helical repeats. Homology between RITS and HUSH is particularly interesting in light of several functional similarities between the two complexes. Both HUSH and RITS affect H3K9me3 deposition over targets, and rely on self-association (of Periphilin or Tas3) to spread this epigenetic mark^24,37^. In RITS H3K9me3 deposition is induced by a positive feedback loop based on recognition of nascent repetitive transcripts. Observations that MPP8 genome binding is enhanced by LINE-1 transcription^15^ and that TASOR and Periphilin are mRNA binding proteins^24–26^ are, therefore, pertinent. The functional relationship between HUSH and RITS is further supported by our epigenomic profiling of TASOR-regulated H3K9me3, which strengthens the notion that HUSH targets for H3K9 methylation in human cells are primarily intronic L1P repeats, or repetitive exons in transcribed genes. It remains unclear how recognition of an RNA intermediate by HUSH and H3K9 methylation of HUSH loci by SETDB1/ATF7IP might be linked. The H3K9me3 mark is not solely spread via a simple read-write mechanism involving MPP8 and SETDB1, as the MPP8 chromodomain is not required to maintain pre-established repression but TASOR and Periphilin are^11^. Another domain, such as the TASOR pseudo-PARP domain, could be required to activate SETDB1. Alternatively, a specific structure or compact physical state of the chromatin, generated by HUSH effector MORC2 (refs. ^13–15^), may be necessary to license target loci for SETDB1 methylation.

HUSH target elements have in common the potential to cause genome damage if incorrectly processed. HUSH may therefore play a role in controlling the rate of transcription of these elements. RNA Polymerase II (Pol II) is known to transcribe H3K9me3-marked and repetitive regions more slowly^51,52^. We have reported elsewhere that Periphilin aggregates through a low-complexity sequence reminiscent of disordered RNA-binding proteins^24^. The formation of Periphilin-RNA aggregates could potentially physically impede transcriptional elongation by Pol II. Our protein–protein interaction screen identified several nuclear RNA processing factors as TASOR binders (Fig. 1). One notable example is RPRD2, another LINE-1 repressor^15^, which regulates transcription by directly binding Pol II^53^. HUSH-dependent H3K9me3 could similarly reduce transcription rates to prevent R-loop formation at genomic regions prone to instability^31^, or to ensure correct mRNA processing (e.g., splicing). Indeed, intronic LINE-1s are thought to function as hubs of transcriptional repression that protect long mammalian introns from improper splicing^54^. Local chromatin compaction by HUSH effector MORC2 may provide an additional protective barrier of repression^14^. Loss of these repressive barriers should cause transcription rates to increase, potentially leading to improper processing and genome instability. This may explain the developmental arrest observed in mouse TASOR mutants at the onset of gastrulation^12^.

A potential role for HUSH in genome protection is consonant with our discovery of a pseudo-PARP domain in TASOR. PARPs are central regulators of genome stability, which can be compromised by inappropriate recombination or retrotransposition. We found that the TASOR pseudo-PARP domain is critical for transgene repression (Fig. 2), despite being dispensable for HUSH assembly. Unlike canonical PARPs, TASOR appears from our crystal structure to be incompetent for catalysis (Figs. 4, 5). There are intriguing functional parallels between TASOR and ZAP (PARP13), which is also catalytically inactive. ZAP binds repetitive RNA sequences^55^, influences the destruction of target RNAs^56^ and inhibits LINE-1 retrotransposition^57^. We have identified that TASOR activity relies on an extended loop unique among the PARP family, and a single point mutation (Y305A) in this loop is sufficient to abolish transgene repression, endogenous LINE-1 restriction and genome-wide H3K9me3 deposition (Fig. 6). Structural studies with larger fragments of HUSH in complex with chromatin or RNA will be necessary to illuminate the basis for this loss of function. In light of our NMR data we speculate that the Y305A mutation inhibits a conformational change within the chromatin-engaged HUSH complex, leading to a loss of H3K9me3 deposition and HUSH-mediated repression. Whether the Y305A mutation inhibits SETDB1 activity directly or indirectly (e.g., via changes to chromatin structure) requires further investigation.

Our findings have implications for how HUSH regulation is established and maintained over newly integrated genetic elements such as retroviruses. This is clinically important in the context of HIV latency and gene therapy^58^. We reported previously that HUSH represses transgenes that integrate into H3K9me3-marked chromatin. However, the requirement for the pseudo-PARP domain and Periphilin self-association^24^ in HUSH function (but not assembly) underscores that H3K9me3 reading and writing is insufficient to explain HUSH activities. Whether HUSH has H3K9me3-independent modes of recruitment to target sequences also remains to be resolved. Silencing of unintegrated murine retroviral DNA by HUSH requires NP220 (ref. ^17^), a DNA- and RNA-binding protein thought to be at least partly sequence-specific for cytidine clusters^59^. We find that NP220 and another matrin-type ZNF (ZNF318) interact with TASOR in the absence of virus, raising the possibility that multiple specific adapters could recruit HUSH in different contexts. We note that although nucleotide sequences are one source of specificity in epigenetic repression, in the case of HUSH, specificity could also arise from RNA structure or the repetitiveness of a nucleotide sequence.

## Methods

### Cell culture

HeLa and HEK293T cells (ECACC) were grown in IMDM or DMEM plus 10% fetal calf serum (FCS) and penicillin/streptomycin (100 U/mL). Cell lines were routinely tested for mycoplasma contamination using the MycoAlert detection kit (Lonza).

### Antibodies

The following primary antibodies were used: rabbit α-TASOR (Atlas HPA006735, RRID:AB_1852384 for ChIP, CUT&Tag and western blot); rabbit α-TASOR (abcam ab224393, for microscopy); rat α-mCherry (Thermo Fisher Scientific, M11217, RRID:AB_2536611); rabbit α-MPP8 (Proteintech, 16796-1-AP, RRID:AB_2266644); rabbit- α-V5 (Abcam, ab27671, RRID:AB_471093); mouse α-Myc (Abcam, ab32, RRID:AB_303599); mouse α-FLAG (Millipore Sigma, F1804, RRID:AB_262044; rabbit α-PPHLN1 (Abcam, ab69569, RRID:AB_1269877); rabbit α-H3K9me3 (abcam ab8898, RRID:AB_306848, for CUT&RUN and CUT&Tag); rabbit α-H3K27me3 (CST C36B11, RRID:AB_2616029, for CUT&RUN positive control); guinea pig α-rabbit IgG (CSB-PA00150E1Gp, for CUT&Run and CUT&Tag); rabbit IgG (CST 2729, RRID:AB_1031062 for ChIP negative control); rabbit α-ORF1p (CST D3W9O, RRID:AB_2800129); mouse α-β-actin (abcam ab8226, RRID:AB_306371). Dilutions were made according to manufacturer recommendations unless otherwise stated.

### Lentiviral expression

Exogenous expression of TASOR variants and other HUSH components was achieved using the lentiviral expression vectors pHRSIN-pSFFV-GFP-WPRE-pPGK-Hygro, pHRSIN-pSFFV-GFP-WPRE-pPGK-Blasto or pHRSIN-pSFFV-GFP-pPGK-Puro with mCherry-TASOR (full-length or mutant), HA-BirA-TASOR (full-length or mutant), V5-PPHLN1 or myc-SETDB1 cassettes inserted in place of GFP. Lentivirus was generated through the triple transfection of HEK 293T cells with the lentiviral transfer vector plus the two packaging plasmids pCMVΔR8.91 and pMD.G using TransIT-293 transfection reagent (Mirus) as recommended by the manufacturer. Viral supernatant was typically harvested 48 h post-transfection, cell debris removed using a 0.45 µm filter, and target cells transduced by spin infection at 800 g for 1 h. Transduced cells were selected with hygromycin (100 µg/mL), blasticidin (5 µg/mL) or puromycin (2 µg/mL).

### Co-immunoprecipitation and western blotting

For co-immunoprecipitation, cells were lysed in 1% NP-40 in TBS plus 10 mM iodoacetamide, 0.5 mM phenylmethylsulfonyl fluoride (PMSF) and benzonase (Sigma-Aldrich) for 30 min. Protein A and IgG-sepharose resin was added to the lysates along with primary antibody. The suspension was incubated for 2 h at 4 °C and the resin was washed three times in lysis buffer. For western blotting, cells were lysed with lysis buffer containing 1% SDS instead of 1% NP-40. For SDS-PAGE analysis, resins or lysates were heated to 70˚C in SDS sample buffer for 10 min and run on a polyacrylamide gel. Gels were blotted onto PVDF membranes (Millipore). Blots were blocked in 5% milk in PBS, 0.2% Tween-20 and incubated with primary antibody diluted in blocking solution. As the Periphilin antibody was unable to detect its epitope under NP-40 lysis conditions, we used a mouse antibody against the V5 tag (Abcam, ab27671) as the primary antibody for Periphilin. For TASOR, the primary antibody was rabbit α-TASOR (Atlas, HPA006735). Blots were imaged with West Pico or West Dura (Thermo Fisher Scientific).

### Flow cytometry

Cells were fixed in 1% PFA and analyzed on a FACSCalibur or a FACSFortessa instrument (BD). Data were analyzed using FlowJo (v10) software. For cell sorting, cells were resuspended in PBS + 2% FCS and an Influx cell sorter (BD) was used.

### CUT&RUN

We followed the protocol detailed by the Henikoff lab^27^. Briefly, 1–2.5 × 10^5^ cells (per antibody/cell line combination) were washed twice (20 mM HEPES pH 7.5, 150 mM NaCl, 0.5 mM spermidine, 1× Roche complete protease inhibitors) and attached to ConA-coated magnetic beads (Bangs Laboratories) that had been pre-activated in binding buffer (20 mM HEPES pH 7.9, 10 mM KCl, 1 mM CaCl_2_, 1 mM MnCl_2_). Cells bound to the beads were resuspended in 50 µL buffer (20 mM HEPES pH 7.5, 0.15 M NaCl, 0.5 mM Spermidine, 1× Roche complete protease inhibitors, 0.02% w/v digitonin, 2 mM EDTA) containing primary antibody (1:100 dilution). Incubation proceeded at 4 °C for at least 2 h (usually overnight) with gentle shaking. Tubes were then placed on a magnet stand to allow removal of unbound antibody, and washed three times with 1 mL digitonin buffer (20 mM HEPES pH 7.5, 150 mM NaCl, 0.5 mM Spermidine, 1× Roche complete protease inhibitors, 0.02% digitonin). After the final wash, pA-MNase (35 ng per tube, a generous gift from Steve Henikoff) was added in a volume of 50 µL of the digitonin buffer and incubated with the bead-bound cells at 4 °C for 1 h. Beads were washed twice, resuspended in 100 µL of digitonin buffer, and chilled to 0–2 °C. Genome cleavage was stimulated by addition of 2 mM CaCl_2_ (final), briefly vortexed and incubated at 0 °C for 30 min. The reaction was quenched by addition of 100 µL 2× stop buffer (0.35 M NaCl, 20 mM EDTA, 4 mM EGTA, 0.02% digitonin, 50 ng/µL glycogen, 50 ng/µL RNase A, 10 fg/µL yeast spike-in DNA (a generous gift from Steve Henikoff)) and vortexing. After 10 min incubation at 37 °C to release genomic fragments, cells and beads were pelleted by centrifugation (16,000 × *g*, 5 min, 4 °C) and fragments from the supernatant purified with a Nucleospin PCR clean-up kit (Macherey-Nagel). Experimental success was evaluated by capillary electrophoresis (Agilent) with this material and the presence of nucleosome ladders for histone modifications (H3K27me3 or H3K9me3) but not for IgG controls. Illumina sequencing libraries were prepared using the Hyperprep kit (KAPA) with unique dual-indexed adapters (KAPA), pooled and sequenced on a HiSeq4000 or NovaSeq6000 instrument. Paired-end reads (2 × 150) were aligned to the human and yeast genomes (hg38 and R64-1-1, respectively) using Bowtie2 (v2.3.4.3; --local –very-sensitive-local –no-mixed –no-discordant –phred33 -I 10 -X 700) and converted to bam files with samtools (v1.9)^60,61^. Conversion to bedgraph format and normalization was done with bedtools genomecov (v2.27.1; -bg -scale), where the scale factor was the inverse of the number of reads mapping to the yeast spike-in genome^62^. Peaks were called in SEACR^63^ (v1.1; stringent, norm options) for experimental samples against IgG controls. The RUVseq package (v1.20) was used to remove unwanted variation prior to differential binding analysis with edgeR (v3.28.1)^64,65^. TASOR-regulated H3K9me3 peaks were defined as those under a cutoff in the MA plot (log_2_ fold-change < −1) of KO vs control cells. CUT&RUN experiments to assess H3K9me3 regulation were done with four replicates (control and TASOR KO cells, used to define TASOR-regulated peaks) or two replicates (WT and Y305A complemented cells, used to assess H3K9me3 complementation over TASOR-regulated peaks). Normalized bigwig files were generated (UCSC), displayed in IGV (v2.4.9)^66^ and heatmaps plotted with deepTools computeMatrix and plotHeatmap commands (v3.3.0). Replicate correlations were assessed with deepTools multiBigWigSummary and plotCorrelation commands^67^ (Supplementary Fig. 6).

### ChIP-seq

Cells (10 million per IP) were washed once in PBS, resuspended in growth medium, and then cross-linked in 1% formaldehyde for 10 min. The reaction was quenched by adding glycine to a final concentration of 0.125 M for 5 min before the cells were lysed in cell lysis solution (10 mM HEPES pH 7.5, 85 mM KCl, 0.5% IGEPAL). Nuclei were pelleted by centrifugation, and then resuspended in nuclear lysis solution (50 mM Tris pH 8.1, 10 mM EDTA, 1% SDS) for 10 min. The chromatin was sheared using a Bioruptor (Diagenode, high power, 20 cycles of 30 s with 30 s recovery) to obtain a mean fragment size of ~300 bp. Insoluble material was removed by centrifugation. The chromatin solution was pre-cleared with protein A sepharose (Sigma-Aldrich), 10% retained (input) and then chromatin immunoprecipitated overnight using 5 µg primary antibody (rabbit IgG or rabbit α-TASOR) and protein A sepharose. The next day the beads were washed a total of five times, and then bound protein-DNA complexes eluted in 0.15 M NaHCO_3_ and 1% SDS. Cross-links were reversed by overnight incubation at 67 °C with 0.3 M NaCl and 1 µg RNase A. Proteinase K (60 µg) was then added and the samples incubated for 2 h at 45 °C. DNA was purified using a spin column (Qiagen PCR purification kit). Illumina sequencing libraries were produced from this material using the TruSeq kit (Illumina), and sequenced on a HiSeq 2500 instrument. Single-end reads (1 × 50) were aligned to the human genome (hg38) using Bowtie2 with default parameters and converted to bam files with samtools^60,61^. Coverage plots for input and ChIP (IgG and TASOR) samples were generated using bamCoverage (deepTools), with reads extended (250 bp) and normalized using RPGC (reads per genomic context; chrX ignored) with an effective genome size of 2913022398 (hg38)^67^. ChIP experiments were done once. Normalized bigwig files were displayed in IGV^66^ and heatmaps generated with deepTools computeMatrix and plotHeatmap commands^67^.

### CUT&Tag

We followed the protocol detailed by the Henikoff lab^28^ with alterations made after consultation with protocol authors, or due to the method being under regular review and optimization. 100,000 cells were washed twice (20 mM HEPES pH 7.5, 0.15 M NaCl, 0.5 mM spermidine, 1× Roche complete protease inhibitors) and attached to activated ConA-coated magnetic beads (Bangs Laboratories) at RT for 15 min. Cells bound to the beads were resuspended in 100 µL buffer (20 mM HEPES pH 7.5, 0.15 M NaCl, 0.5 mM Spermidine, 1× Roche complete protease inhibitors, 0.05% digitonin (Millipore), 2 mM EDTA) containing primary antibody (1:50 dilution). Incubation proceeded at RT for 2 h with gentle shaking. Tubes were placed on a magnet stand to allow removal of unbound antibody. The secondary antibody (guinea pig anti-rabbit IgG, 0.25 g/L) was added at 1:100 dilution and cells incubated at RT for 1 h with gentle shaking. Cells were washed three times on the magnet in 1 mL buffer (20 mM HEPES pH 7.5, 150 mM NaCl, 0.5 mM Spermidine, 1× Roche complete protease inhibitors, 0.05% digitonin). Meanwhile pA-Tn5 adapter complex (40 nM, a generous gift from Steve Henikoff) was prepared in a higher salt, lower digitonin buffer (20 mM HEPES, pH 7.5, 0.35 M NaCl, 0.5 mM spermidine, 1× Roche complete protease inhibitors, 0.01% digitonin). This buffer had a slightly increased NaCl concentration over that recommended in the protocol, to reduce non-specific binding to open chromatin (the so-called ATAC-seq artifact). After the final wash, 100 µL of pA-Tn5 solution was added to the bead-bound cells with gentle vortexing and the cells incubated at RT for 1 h with gentle shaking. Cells were then washed three further times in 1 mL buffer, before resuspension in 50 µL tagmentation buffer (20 mM HEPES pH 7.5, 0.35 M NaCl, 10 mM MgCl_2_, 0.5 mM spermidine, 1× Roche complete protease inhibitors, 0.01% digitonin). Tagmentation was allowed to proceed at 37 °C for 1 h before quenching with 20 mM EDTA, 0.5% SDS (both final concentrations) and 10 µg Proteinase K (Thermo Fisher Scientific). The mixture was incubated at 37 °C overnight. The next day, tubes were incubated at 70 °C for 20 min to further inactivate the protease. DNA was extracted from the mixture with 2.2× SPRI beads (KAPA). After twice washing the beads with 80% EtOH, DNA was eluted in 25 µL water. For PCR, 21 µL DNA was mixed with 2 µL of 10 µM universal i5 primer (AATGATACGGCGACCACCGAGATCTACACTCGTCGGCAGCGTCAGATGTG) and 2 µL of 10 µM uniquely-barcoded i7 primer (CAAGCAGAAGACGGCATACGAGAT[i7]GTCTCGTGGGCTCGGAGATGT), then 25 µL NEBNext HiFi 2× PCR Master mix was added and pipette mixed. The following thermocycler program was used: 72 °C for 5 min; 98 °C for 30 s; 13 cycles of 98 °C for 10 s and 63 °C for 30 s; final extension at 72 °C for 1 min and hold at 8 °C. Post-PCR clean-up was performed with 1.1× SPRI beads (KAPA). Libraries were pooled in approximately equimolar ratios based on capillary electrophoresis (Agilent) and/or fluorometry (Thermo Fisher Scientific) quantification results, before final left-sided size selection (1.1× SPRI) to remove residual PCR primers. Paired-end reads (2×150 bp) were generated on a HiSeq 4000 instrument (Illumina). Reads were aligned to the human genome (hg38) using Bowtie2 (--local –very-sensitive-local –no-mixed –no-discordant -I 10 -X 700) and converted to bam files with samtools^60,61^. PCR duplicates were removed using Picard (http://broadinstitute.github.io/picard/) before conversion to bedgraph file format (bedtools)^62^. Coverage plots for display and comparison of tracks were generated using bamCoverage (deepTools)^67^ after downsampling bam files to the control/KO sample based on the final number of mapped reads. We note that this is a conservative approach, because library complexity is related to the number of true binding sites in such a targeted experiment. CUT&Tag experiments to assess TASOR binding were done in biological duplicate. Resulting bigwig files were displayed in IGV^66^ and heatmaps made with deepTools computeMatrix and plotHeatmap packages. Replicate correlations were assessed with deepTools multiBigWigSummary and plotCorrelation commands^67^ (Supplementary Fig. 6).

### RNA-seq analysis

Paired-end (2 × 150) reads from HeLa RNA-seq experiments that we reported elsewhere^14^ were re-mapped to hg38 using HiSat2 (v2.1.0; --no-mixed –no-discordant) then converted to bam files in samtools^61,68^. Fragments overlapping representative transcripts from annotated human genes (gencode v29) were counted in the featureCounts tool (--primary –fraction -t exon -p) from the subread package (v1.6.4)^69^ and the mean taken of the three replicates.

### BioID

The following protocol was adapted from a published protocol^70^. TASOR knockout cells expressing HA-BirA-tagged TASOR variants were grown in square 500 cm^2^ dishes (Corning) in DMEM, which was supplemented with 50 μM biotin for 18 h prior to downstream processing. After washing with PBS, cells were scraped in PBS before pelleting (400 ×  *g*, 5 min, RT). Nuclei were isolated by resuspending cells in 10 mL nuclear isolation buffer (1 mM HEPES, 85 mM KCl, 0.5% IGEPAL) before being pelleted again (800 × *g*, 5 min, 4 °C). Nuclear pellets were lysed by sonication on ice in 10 mL lysis buffer (50 mM Tris pH 7.4, 0.5 M NaCl, 0.4% SDS, 5 mM EDTA, 1 mM DTT, 1× Roche complete protease inhibitor). After sonication, Triton X-100 concentration was adjusted to 2% and NaCl concentration to 150 mM. Lysates were then centrifuged (16,000 × *g*, 10 min, 4 °C). Protein concentration was measured by BCA assay (Pierce) and equal amounts taken for further steps. Samples were incubated with 50 μL pre-equilibrated magnetic Dynabeads MyOne Streptavidin C1 at 4 °C overnight on a rotating wheel. The next day, a series of washing steps were carried out at RT, each twice for 5 min unless otherwise stated. Wash buffer 1 contained 2% SDS, 50 mM TEAB pH 8.5, 10 mM TCEP, 20 mM iodoacetamide (30 min incubation). Wash buffer 2 contained 50 mM HEPES pH 7.4, 1 mM EDTA, 500 mM NaCl, 1% Triton X-100, 0.1% Na-deoxycholate. Wash buffer 3 contained 10 mM Tris pH 8.0, 0.25 M LiCl, 1 mM EDTA, 0.5% NP-40, 0.5% Na-deoxycholate. Wash buffer 4 contained 50 mM Tris pH 7.4, 50 mM NaCl, 0.1% NP-40. Wash buffer 5 contained 50 mM TEAB pH 8.5, 6 M urea. Wash buffer 6 contained 50 mM TEAB pH 8.5. Finally, beads were resuspended in 50 µL 50 mM TEAB pH 8.5 containing 0.1% Na-deoxycholate and 200 ng of trypsin (Promega), before incubation overnight at 37 °C with periodic shaking (Eppendorf Thermomixer). After removing the beads digests were subjected to clean-up using SDP-RPS extraction material (Affinisep) backed into 200 µL pipette tips. Columns were conditioned with 100 µL ACN followed by 100 µL 0.5% TFA. Samples were acidified with a final concentration of 0.5% TFA and an equal volume of ethyl acetate loaded onto the columns. Columns were then washed with 100 µL 0.2% TFA and 100 µL ethyl acetate and eluted in 80% ACN + 5% ammonium hydroxide. Samples were dried under vacuum and stored at −20 °C prior to analysis.

### Mass spectrometry

Samples were resuspended in 10 µL 5% DMSO, 0.5% TFA and the whole sample injected. Data were acquired on an Orbitrap Fusion mass spectrometer (Thermo Scientific) coupled to an Ultimate 3000 RSLC nano UHPLC system (Thermo Scientific). Samples were loaded at 10 μL/min for 5 min on to an Acclaim PepMap C18 cartridge trap column (300 µm × 5 mm, 5 µm particle size) in 0.1% TFA. After loading, a linear gradient of 3–32% solvent B over 60 min was used for sample separation with a column of the same stationary phase (75 µm × 75 cm, 2 µm particle size) before washing at 90% B and re-equilibration. Solvents were A: 0.1% FA and B: ACN/0.1% FA. MS settings were as follows. MS1: quadrupole isolation, 120,000 resolution, 5e5 AGC target, 50 ms maximum injection time, ions accumulated for all parallelisable time. MS2: quadrupole isolation at an isolation width of *m/z* 0.7, HCD fragmentation (NCE 34) with the ion trap scanning out in rapid mode from, 8e3 AGC target, 0.25 s maximum injection time, ions accumulated for all parallelisable time. Target cycle time was 2 s. Spectra were searched by Mascot within Proteome Discoverer 2.2 in two rounds of searching. The first search was against the Uniprot human reference proteome and compendium of common contaminants (GPM). The second search took all unmatched spectra from the first search and searched against the human trEMBL database. The following search parameters were used. MS1 Tol: 10 ppm, MS2 Tol: 0.6 Da, fixed mods: carbamindomethyl (C); var mods: oxidation (M), enzyme: trypsin (/P). Peptide spectrum match (PSM) FDR was calculated using Mascot percolator and was controlled at 0.01% for ‘high’ confidence PSMs and 0.05% for ‘medium’ confidence PSMs. Proteins were quantified using the Minora feature detector within Proteome Discoverer.

### Western blotting

Cells were lysed in 1% SDS plus 1:100 (v/v) benzonase (Sigma) for 15 min at room temperature, and then heated to 65 °C in SDS sample loading buffer for 5 min. Following separation by SDS-PAGE, proteins were transferred to a PVDF membrane (Millipore), which was then blocked in 5% milk in PBS + 0.2% Tween-20. Membranes were probed overnight with the indicated primary antibodies, washed four times in PBS + 0.2% Tween-20, then incubated with HRP-conjugated secondary antibodies for 1 h at RT. Reactive bands were visualized using SuperSignal West Pico (Thermo Fisher Scientific). Alternatively, after SDS-PAGE, proteins were transferred to a nitrocellulose membrane (Thermo Fisher Scientific iBlot2), which was blocked in 5% milk in PBS (no detergent) for 1 h. Membranes were probed overnight with the indicated primary antibodies in 5% milk in PBS + 0.1% Tween-20, washed thoroughly in PBS + 0.1% Tween-20, then incubated with DyLight-680 or 800-conjugated secondary antibodies (Thermo Fisher Scientific) at 1:10,000 dilution for 30 min at RT. After thorough washing with PBS-Tween, PBS and then water, blots were imaged on the Odyssey near-infrared system (LI-COR). Uncropped blots are shown in Supplementary Fig. 7.

### Subcellular fractionation

Cells were washed twice in PBS and once in buffer A (10 mM HEPES pH 7.9, 1.5 mM MgCl_2_, 10 mM KCl, 0.5 mM dithiothreitol (DTT) and protease inhibitor cocktail). Cells were then pelleted and resuspended in buffer A with 0.1% (v/v) NP40 and incubated on ice for 10 min. The supernatant containing the cytoplasmic fraction was collected following centrifugation (1300 × *g*, 4 min, 4 °C) and further clarified by high-speed centrifugation (20,000 × *g*, 15 min, 4 °C). The remaining pellet was washed in buffer A without NP40 and resuspended in an equal volume (relative to the cytoplasmic extract) of buffer B (20 mM HEPES pH 7.9, 1.5 mM MgCl_2_, 0.3 M NaCl, 0.5 mM DTT, 25% (v/v) glycerol, 0.25% Triton X-100, 0.2 mM EDTA and protease inhibitor cocktail). The supernatant containing the soluble nuclear fraction was collected following centrifugation (1,700 g, 4 min, 4 °C), and the insoluble pellet, composed primarily of chromatin and associated proteins, was resuspended in an equal volume of Laemmli buffer (relative to the cytoplasmic and soluble nuclear extracts). Equal volumes of cytoplasmic, soluble and insoluble nuclear fractions were separated by SDS-PAGE, transferred to a PVDF membrane (Millipore) and probed with relevant antibodies.

### Confocal immunofluorescence microscopy

Cells were fixed for 15 min with 4% formaldehyde in PBS, permeabilized for 5 min with 0.1% TritonX-100 in PBS and then blocked with 2% BSA in PBS for 1 hour. Cells were stained with primary antibodies at 1:200 dilution in blocking buffer and, after further washing, with secondary antibody (anti-rabbit AlexaFluor 647, 1:500 dilution) in blocking buffer for 1 h. Samples were washed thoroughly and cover slips mounted on microscopy glasses with ProLong Gold anti-fade reagent with DAPI (Invitrogen). Imaging was performed using Nikon Ti microscope equipped with CSU-X1 spinning disc confocal head (Yokogawa) and with Zeiss 780 system.

### Protein expression and purification

A synthetic *E. coli* codon-optimized DNA construct (IDT) encoding TASOR residues 106–332 (UniProt Q9UK61-1) was cloned into the expression vector pET-15b for production of the N-terminally thrombin-cleavable His_6_-tagged protein product (MGSSHHHHHHSSGLVPRGSHM[…]). Mutation of this construct to generate variants Y305A or the construct used for crystallography (110–332 Δ261–269) were done with standard methods. Transformed *E. coli* BL21(DE3) cells (NEB) were grown at 37 °C in 2xTY media containing 100 mg/L ampicillin. Expression was induced at an OD_600_ of 0.8 with 0.2 mM IPTG for 18 h at 18 °C. The culture was pelleted and resuspended in a buffer containing 50 mM Tris pH 8.0, 0.15 M NaCl, 10 mM imidazole, 1 mM DTT and 1× Roche complete EDTA-free protease inhibitors, then flash frozen in liquid nitrogen and stored at −80 °C. All subsequent steps were done at 4 °C unless otherwise stated. Further lysis was achieved by extensive sonication (3 × 3 min). A solution of benzonase (1:10,000 v/v final concentration, Sigma) was added and after 30 min incubation with stirring, the NaCl concentration was adjusted to 0.5 M, otherwise the protein co-purified with host RNA. The lysate was clarified by centrifugation (15,000 × *g*, 45 min) and the protein-containing supernatant bound to preequilibrated Ni-NTA beads (Generon) for 1 h with rocking. The beads were washed with at least 20 CV Ni wash buffer (50 mM Tris, pH 8.0, 0.5 M NaCl, 10 mM imidazole, 1 mM DTT) before a stepwise elution in batch mode with 3 × 5 CV of Ni wash buffer supplemented with 0.2 M, 0.3 M and 0.5 M imidazole. Further purification was achieved with size-exclusion chromatography on a Superdex 200 increase (10/300) column (GE) in buffer (50 mM HEPES, pH 7.5, 0.2 M NaCl, 0.5 mM TCEP). For crystallography trials and NMR experiments, the His_6_-tag was cleaved with restriction-grade thrombin (Millipore) overnight on ice in Tris buffer supplemented with 2.5 mM CaCl_2_. The next day the protease was removed by incubation with 100 µL benzamidine sepharose beads (GE) for 5 min at RT. For crystallography trials an additional ion exchange chromatography step with a monoS column was included in the protocol. Tag cleavage was done after the Ni-affinity step, before size exclusion and ion exchange chromatography steps. For SeMet-labeled protein, expression was repeated in minimal media containing L-(+)-selenomethionine (Anatrace), using an established strategy^71^. Single ^15^N labeling, double ^13^C/^15^N labeling or triple ^2^H/^13^C/^15^N labeling for NMR experiments required expression in minimal media made with ^15^NH_4_Cl, ^13^C glucose and/or D_2_O (Sigma) as appropriate. Cultures in D_2_O grew more slowly and were therefore kept at 25 °C throughout. Purification of these labeled samples otherwise followed the protocol used for unlabeled samples.

For co-expression of the TASOR-MPP8 complex in *E. coli*, we co-transformed BL21(DE3) cells with the pET15b vector harboring His-tagged TASOR(354–633) and the pRSF vector harboring MPP8(527-860) (UniProt Q9UK61-1, Q99549-1). The proteins were expressed by growing the cells in autoinduction media for 60 h at 18 °C, and the complex purified by Ni-affinity purification. Eluates were concentrated and analyzed by SEC-MALS at 293 K using a Superdex 200 (10/300) column in a buffer containing 20 mM HEPES pH 7.5, 0.5 M NaCl, 0.5 mM TCEP. Light scattering analysis was performed in the ASTRA software package (Wyatt), using band broadening parameters obtained from a BSA standard run on the same day under identical conditions. MALS data were used to fit the average molar mass across the complex peak (quoted to the nearest kDa).

Expression of full-length His-tagged *Hs*PARP-1 and the in vitro gel-based PARylation assay followed a detailed protocol published elsewhere^72^.

### NMR

NMR data were collected at 298 K using a Bruker Avance II+ 700 MHz spectrometer with triple resonance cryoprobe unless otherwise stated. All samples were prepared with 5% D_2_O as a lock solvent, in PBS (pH 7.0) supplemented with 1 mM TCEP and 0.05% w/v NaN_3_, and degassed prior to data acquisition. ^1^H-^15^N BEST-TROSY (band selective excitation short transient transverse relaxation optimized spectroscopy) spectra were collected for the ^15^N labeled WT TASOR sample and the ^2^H/^13^C/^15^N WT TASOR sample using an optimized pulse sequence^73^. An initial, incomplete assignment of WT TASOR was carried out using standard TROSY based triple resonance spectra with deuterium decoupling: trHNCO and trHNCACO with 2048*64*128 complex points in the ^1^H, ^15^N and ^13^C dimensions respectively, trHNCA and trHNCOCA with 2018*64*160 complex points in the ^1^H, ^15^N and ^13^C dimensions respectively and trHNCACB and trHNCOCACB with 2048*64*110 complex points in the ^1^H, ^15^N and ^13^C dimensions, respectively. This assignment revealed a subset of residues without peak data. A comparison of 2D projections with a limited number of equivalent triple resonance experiments collected on a ^15^N/^13^C only labeled sample revealed additional peak data. This indicated that the deuterated sample had incomplete back exchange of the solvent exchangeable backbone NH protons within the core of the protein. Partial denaturation of the ^13^C/^15^N/^2^H sample with 3.5 M urea in PBS, followed by incremental stepwise dilution of urea back to 0 M, allowed back-exchange and additional data sets were collected to complete the assignment. These additional experiments included trHNCACB, trHNCA, trHNCACO, trHNCO, and trHNCOCA spectra recorded as above. All triple resonance data sets were collected with 20–40% Non-Uniform Sampling (NUS) and processed using compressed sensing^74^. All 2D data sets were processed using Topspin version 3.1 or higher (Bruker) and all spectra analyzed using Sparky 3.115. The assignment was completed for 184/221 non-proline backbone amide resonances using MARS^75^. The dynamic properties of TASOR were investigated using standard Bruker T_1_ and T_2_ relaxation pulse programs. T_1_ and T_2_ data sets were collected on an Avance III HD 800 MHz spectrometer fitted with a triple resonance cryoprobe and T_1_ delays of 50, 100, 200, 500, 800, 1500, 2200, and 3000 ms and T_2_ delays of 16, 32, 48, 64, 96, 128, 160, and 192 ms. Signal intensity measurements and slope fitting was completed using Sparky. A second, higher-resolution T_2_ data set was collected at 950 MHz (Bruker Avance III HD) using the same relaxation delays.

### X-ray crystallography

TASOR PARP domain (residues 110–332 with internal deletion of residues 261–269 and His_6_ tag cleaved), native or SeMet-labeled, was concentrated to 8 g/L (320 µM) in buffer containing 20 mM HEPES pH 7.5, 0.15 M NaCl, 0.5 mM TCEP. Crystals were grown at 18 °C by the sitting drop vapor diffusion method, by mixing the protein at a 1:1 ratio with reservoir solution containing 0.1 M MES pH 6.5, 0.1 M NaP_i_, 0.1 M KP_i_, 2 M NaCl. Crystals appeared in overnight and were frozen within 2 d in liquid N_2_ using paraffin oil as a cryoprotectant. X-ray diffraction data were collected at 100 K at Diamond Light Source beamlines i02 and i03. Native and selenium-substituted data sets were collected with X-ray wavelengths of 0.97949 Å and 0.97980 Å, respectively. Data sets were processed using autoproc or xia2 packages^76,77^. Automatic experimental phasing was done in AutoSol (Phenix)^78^ using the single anomalous dispersion (SAD) method with selenium as the heavy atom. The resulting model was built and refined in Coot^79^ and Phenix, before being used as a search model for the native data set in Phaser^80^. Further model building and refinement were done with this data set in Coot and Phenix. The final atomic model had no Ramachandran torsion angle outliers and 98% of torsion angles in favored positions. Crystallographic data are summarized in Table S2.

### Fluorescence polarization

TASOR(106–332) was titrated into RNA-binding buffer (20 mM HEPES pH 7.5, 50 mM NaCl, 2 mM MgCl_2_, 0.5 mM TCEP) containing 33 nM of a single-stranded RNA oligonucleotides 5′-labeled with 6-carboxyfluorescein (6-FAM). Fluorescence polarization of 30-µl samples was measured in 384-well black, clear-bottomed plates (Corning) with a ClarioSTAR plate reader using 482/530 nm filters.

### Differential scanning fluorimetry (DSF)

10 µL samples containing 10 µM protein (TASOR variants or PARP1 catalytic domain) in the presence or absence of benzamide (Sigma) were loaded into glass capillaries (Nanotemper) by capillary action. Intrinsic protein fluorescence at 330 nm and 350 nm was monitored between 15 and 90 °C in the Prometheus NT.48 instrument (Nanotemper), and the T_m_ values calculated within the accompanying software by taking the turning point of the first derivative of the F350:F330 ratio as a function of temperature.

### Reporting summary

Further information on research design is available in the Nature Research Reporting Summary linked to this article.

## Supplementary information

Supplementary Information

Peer Review

Reporting Summary

Description of Additional Supplementary Files

Supplementary Data 1

## Data Availability

The NMR data were deposited in the Biological Magnetic Resonance Bank, data set ID 50094. The structure factors and atomic coordinates for the crystal structure were deposited in the Protein Data Bank (PDB) with code 6TL1. The original experimental X-ray diffraction images were deposited in the SBGrid Data Bank (data.SBGrid.org), with Data ID 742. The CUT&RUN, ChIP and CUT&Tag data generated and analyzed here have been deposited in the Gene Expression Omnibus (GEO) database under accession codes GSE155693 and GSE95480. Source data are provided with this paper.

## Source data

Source Data file
