# Supplementary material for: TASOR is a pseudo-PARP that directs HUSH complex assembly and epigenetic transposon control
Source: Nat Commun. 2020 Oct 2;11:4940. doi: 10.1038/s41467-020-18761-6 (PMC7532188; doi:10.1038/s41467-020-18761-6)
Supplement: Supplementary file 1 — Supplementary Information [file 41467_2020_18761_MOESM1_ESM.pdf]

TASOR is a pseudo-PARP that directs HUSH complex assembly and epigenetic transposon control

Douse et al.

Supplementary Information

Supplementary Figures 1-7

Supplementary Table 1

Supplementary Figures

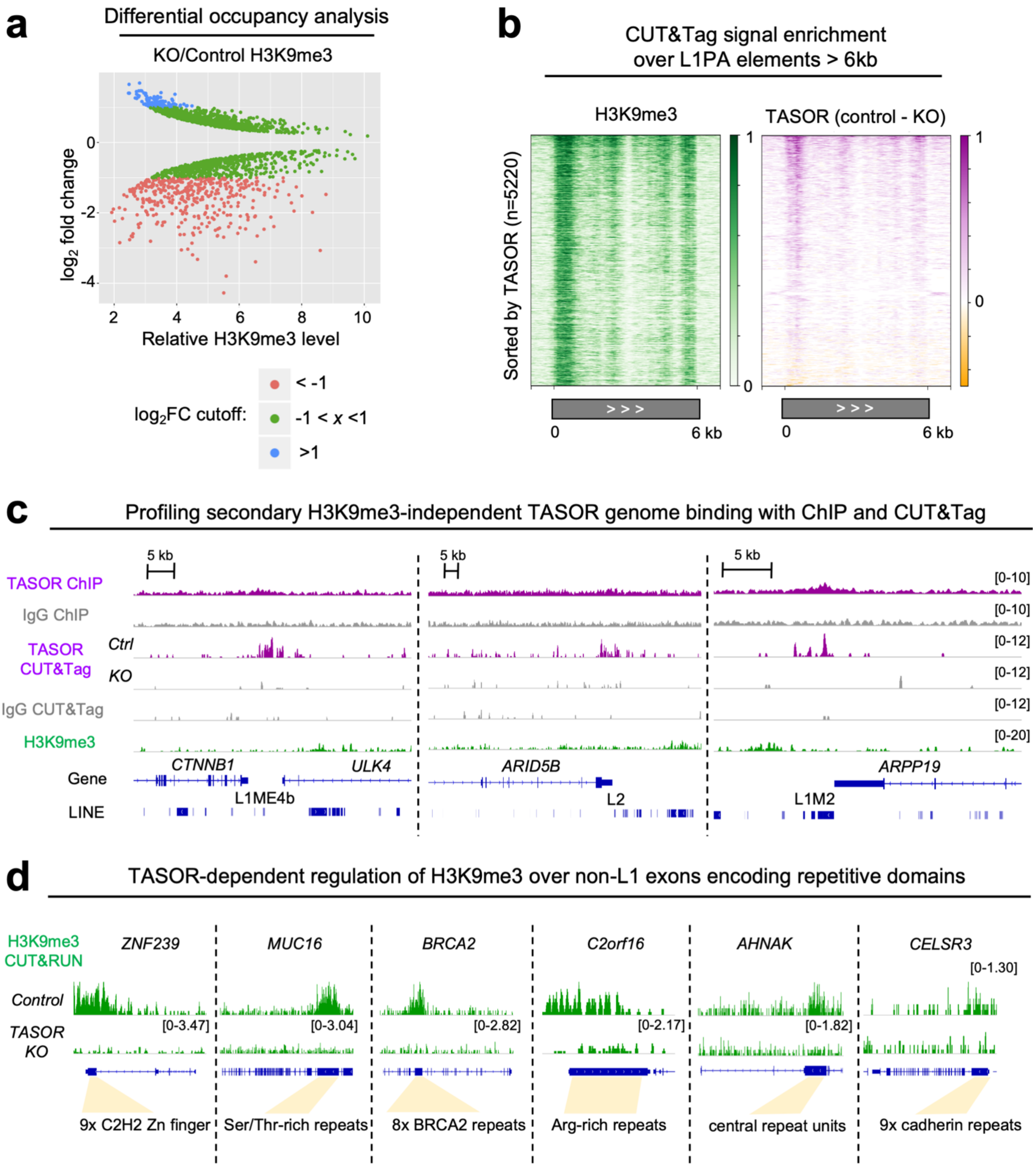

Supplementary Fig. 1. Epigenomic profiling analyses. (**a**) A $\log_2$-fold change <-1 of normalized H3K9me3 counts for TASOR-negative cells versus TASOR-positive cells, and FDR <0.05, was used to define 393 TASOR-regulated sites. (**b**) CUT&Tag H3K9me3 and TASOR signal plotted over 5,220 L1PA elements longer than 6 kb in the hg38 assembly. (**c**) Snapshots showing three loci with evidence of H3K9me3-independent binding by TASOR using ChIP and CUT&Tag. (**d**) Genome browser snapshots showing TASOR-dependent H3K9me3 regulation over repetitive exons that were not LINE-1 elements.

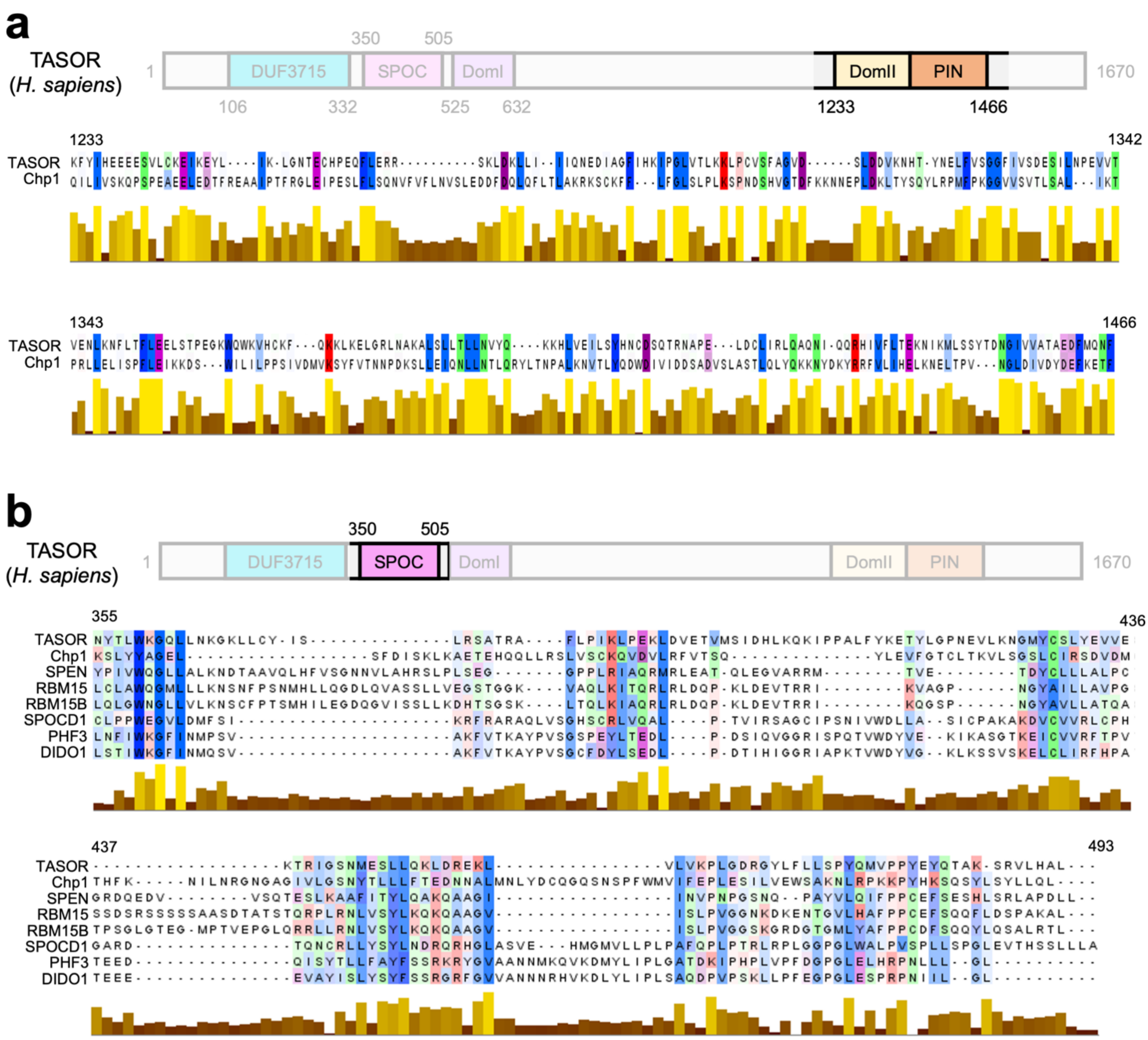

Supplementary Fig. 2. Sequence alignments. (**a**) Sequence alignment of the DomII/PIN domains from TASOR and *S. pombe* Chp1. (**b**) Sequence alignment of SPOC domains from TASOR, Chp1 and six other SPOC-containing proteins.

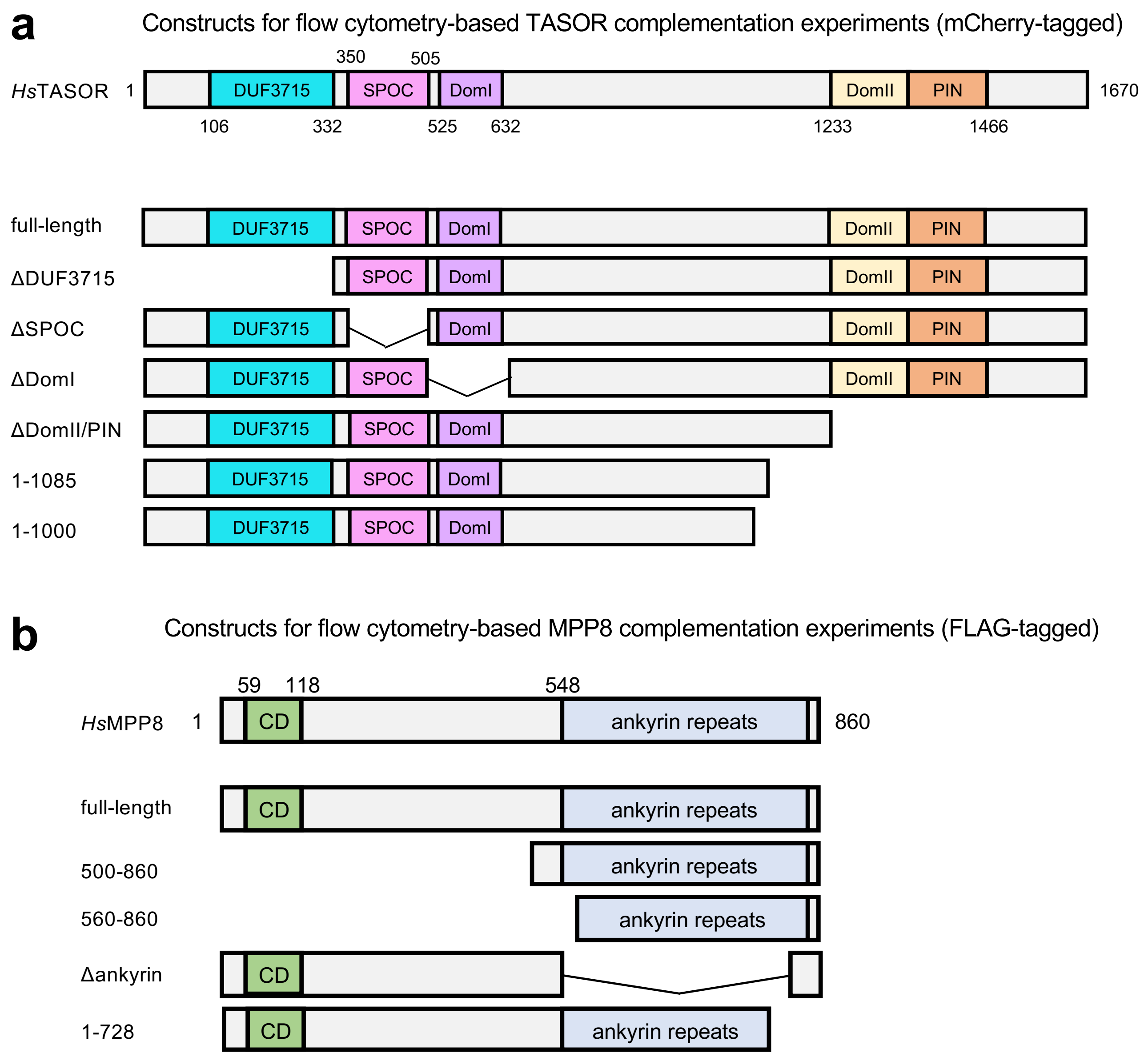

Supplementary Fig. 3. Domain deletion constructs used in function assays. (**a**) Summary of TASOR domain deletion constructs. (**b**) Summary of MPP8 domain deletion constructs for flow cytometry-based transgene repression assays.

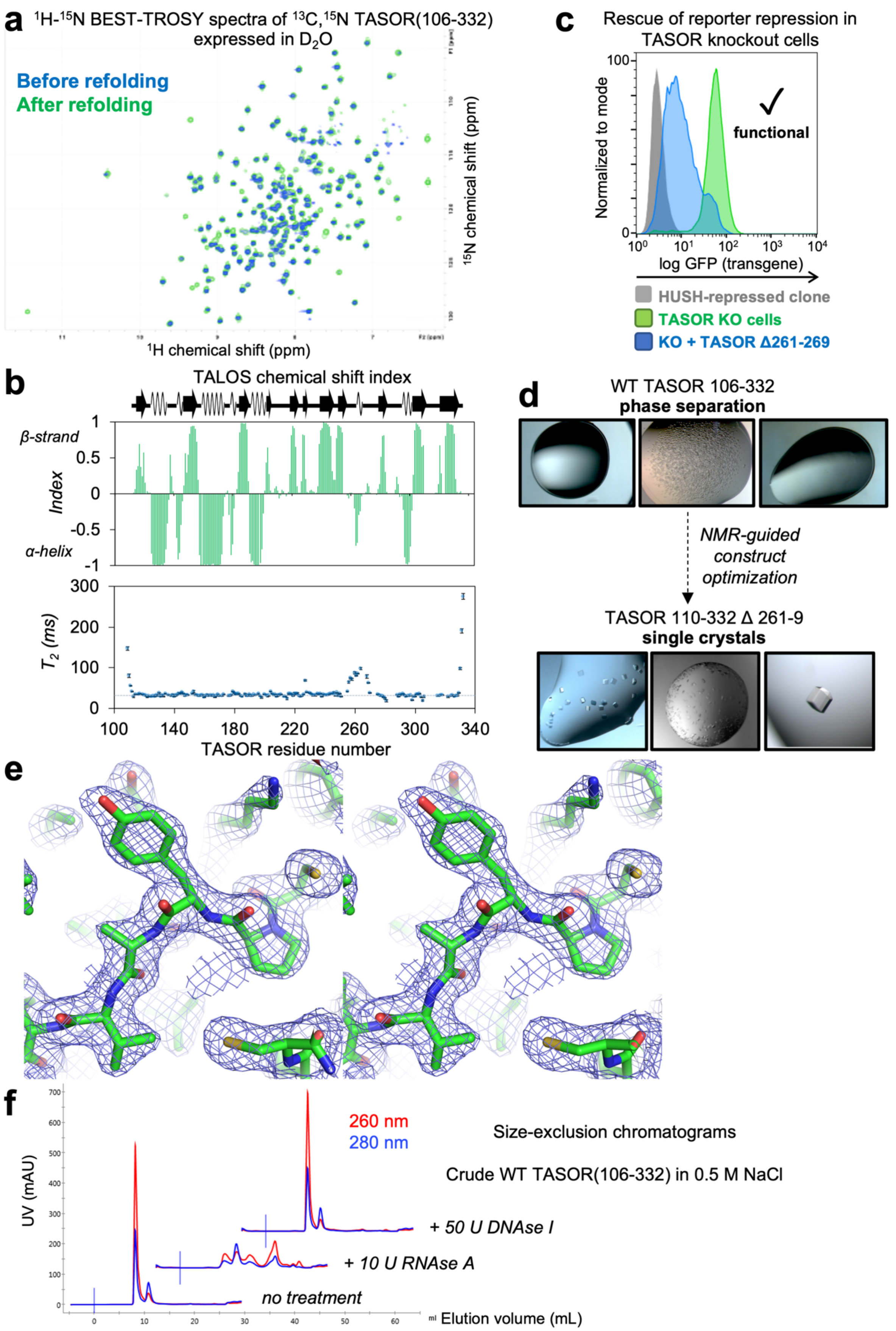

Supplementary Fig. 4. Biophysical analyses of the TASOR PARP (DUF3715) domain. (**a**) NMR spectra showing denaturation/refolding experiment to enforce core amide H/D exchange. (**b**) Chemical shift indexing. Source data provided in a Source Data file. (**c**) FACS transgene repression assay showing 261-269 loop deletion does not affect TASOR function. (**d**) Construct optimization for crystallization. (**e**) Stereo diagram of representative electron density for the crystal structure. (**f**) Superdex 200 column traces following nuclease treatments. TASOR PARP domain coelutes with expression host RNA but not DNA.

a

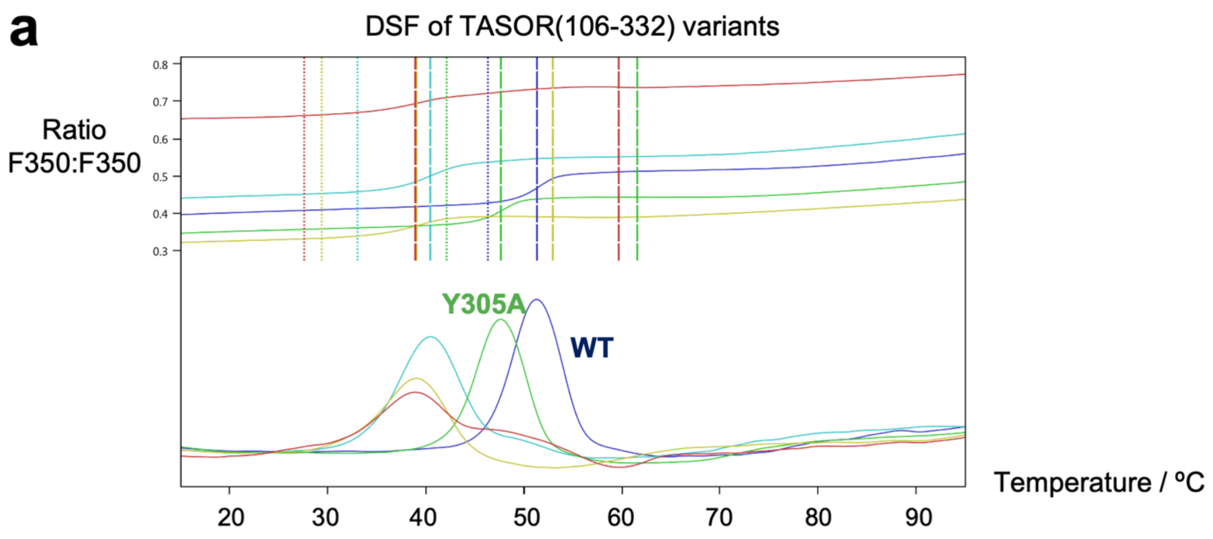

b

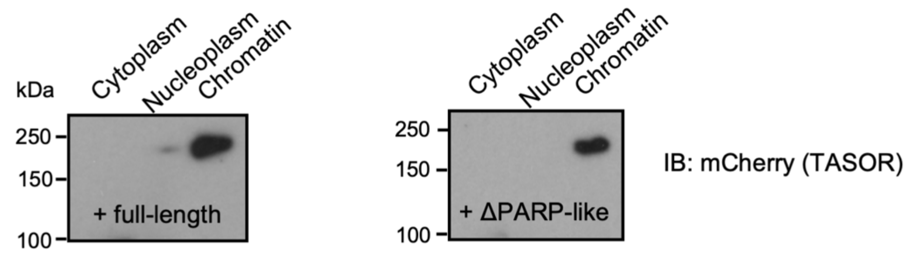

c

		Number of unique peptides		
**Protein name**	**ID**	**WT**	**dPARP**	**Y305A**
Protein TASOR	FAM208A	83	103	107
Neuroblast differentiation-associated protein AHNAK	AHNAK	20	51	33
Zinc finger protein 638	ZNF638	11	65	38
Regulation of nuclear pre-mRNA domain-containing protein 2	RPRD2	7	33	27
M-phase phosphoprotein 8	MPHOSPH8	6	21	22
TOX high mobility group box family member 4	TOX4	6	17	12
Periphilin-1	PPHLN1	5	23	17
Zinc finger protein 318	ZNF318	5	94	42
YLP motif-containing protein 1	YLPM1	4	37	24
Serine/threonine-protein phosphatase 1 regulatory subunit 10	PPP1R10	3	22	17
Cyclin-K	CCNK	2	5	6
Pre-mRNA 3'-end-processing factor FIP1	FIP1L1	2	7	5
Src substrate cortactin	CTTN	2	18	12

Supplementary Fig. 5. Functional consequences of the TASOR Y305A mutation or PARP deletion. (**a**) Differential scanning fluorimetry of WT and Y305A TASOR(106-332) shows both proteins are folded. The upper panel shows the ratio of fluorescence at 350 nm and 330 nm (excitation: 280 nm); the lower panel shows the first derivative of this ratio as a function of temperature (*x* axis). A turning point in the derivative trace defines the melting temperature (T_m) of the domain. (**b**) Subcellular fractionation of full-length TASOR and a PARP deletion construct. (**c**) BioID hits for full-length (WT), PARP deletion and Y305A TASOR variants. Listed are those hits with more than two unique peptides in the WT experiment.

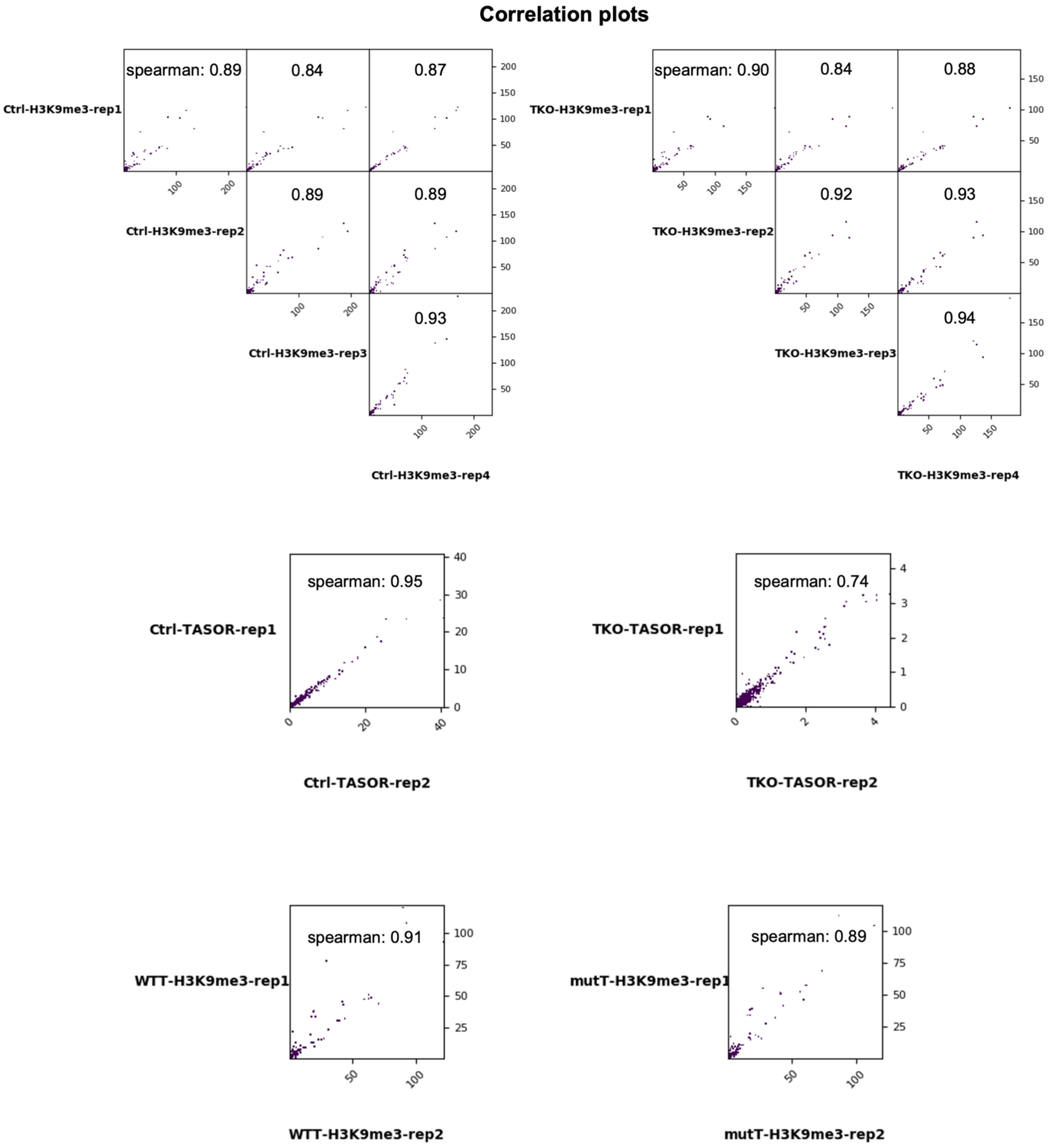

Supplementary Fig. 6. Correlation plots for epigenomic profiling replicates. Related to Fig. 1 and Fig. 6. Four biological replicates were performed for H3K9me3 CUT&RUN in control (Ctrl) and TASOR KO (TKO) cells. Two biological replicates were performed for TASOR CUT&Tag in control (Ctrl) and TASOR KO (TKO) cells. Two replicates were performed for H3K9me3 CUT&RUN in TASOR KO cells complemented with WT TASOR (WTT) or Y305A TASOR (mutT).

Western blots

Fig. 3a

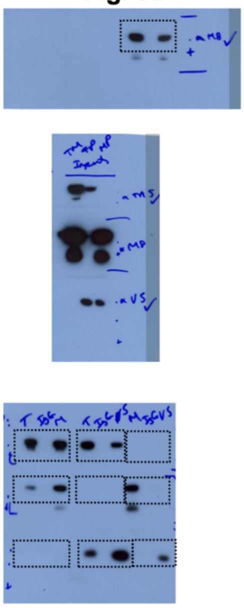

Fig. 3b

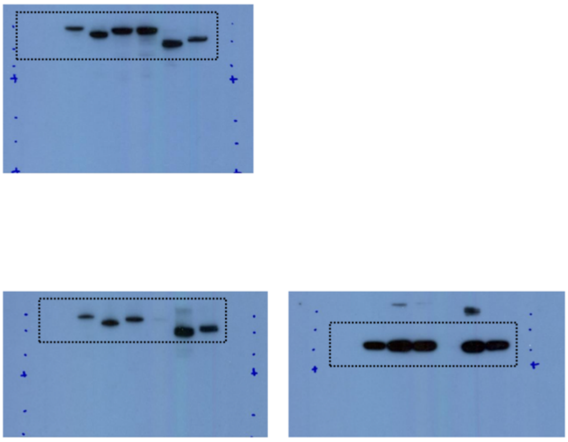

Fig. 3d

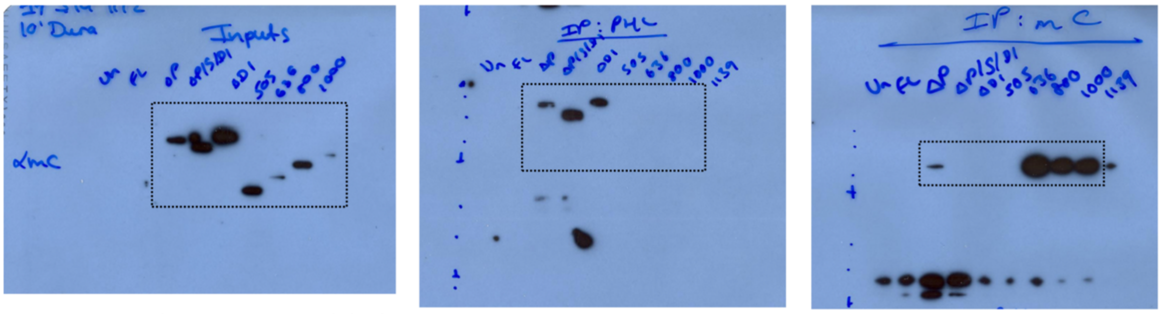

Fig. 6d (two-colour LICOR)

Channel 1 Channel 2

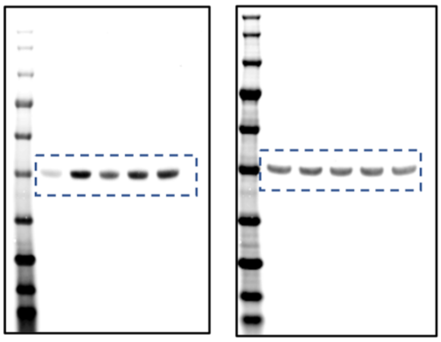

Supplementary Fig. 5b

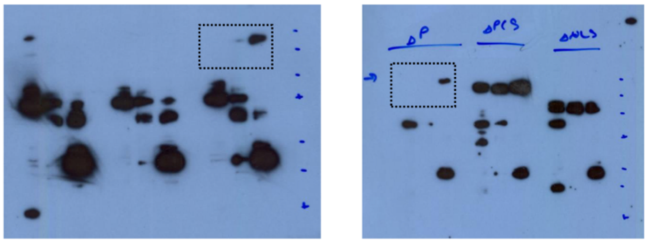

SDS-PAGE (Coomassie-stained)

Fig. 3c

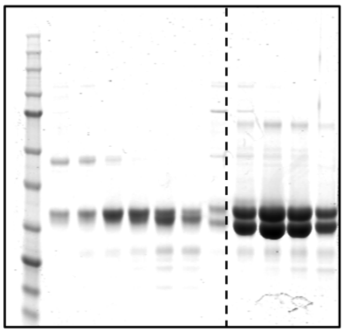

Fig. 5d

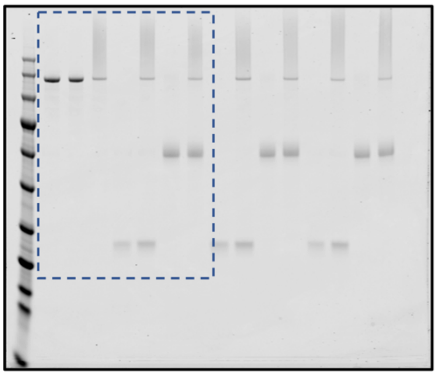

Supplementary Fig. 7. Uncropped Western blots and Coomassie-stained SDS-PAGE gels.

Supplementary Tables

Supplementary Table 1. Crystallographic data collection and refinement statistics for TASOR pseudo-PARP domain.

Dataset	Native	Se-SAD
**Data collection**		
X-ray source	DLS i02	DLS i03
Space group	$P4_12_12$	$P4_12_12$
Cell dimensions		
a = b, c (Å)	74.59, 184.0	74.45, 182.3
α = β = γ (°)	90	90
Resolution (Å)	57.94 – 2.03 (2.08 – 2.03)*	91.14 – 2.27 (2.39 – 2.27)*
Observations	438905	350588
Unique reflections	34589	24704
R_{merge}	0.094 (1.460)	0.115 (1.296)
$\langle I \rangle / \sigma(I)$	16.1 (2.1)	18.4 (2.5)
Completeness (%)	100.0 (100.0)	100.0 (100.0)
Redundancy	12.7 (12.6)	14.2 (14.4)
CC_{ano}	–	0.846 (0.008)
**Refinement**		
Protein molecules in a.u.	2	
R_{work} / R_{free}	0.195 / 0.229	
No. of non-H atoms		
Protein	3359	
Solvent	146	
Mean B-factors		
Protein	56.6	
Solvent	49.3	
R.m.s deviations		
Bond lengths (Å)	0.007	
Bond angles (°)	1.02	

*Highest resolution shell is shown in parentheses.
A.u, asymmetric unit; R.m.s., root mean square.
