# Supplementary material for: TASOR is a pseudo-PARP that directs HUSH complex assembly and epigenetic transposon control
Source: Nat Commun. 2020 Oct 2;11:4940. doi: 10.1038/s41467-020-18761-6 (PMC7532188; doi:10.1038/s41467-020-18761-6)
Supplement: Supplementary file 3 — Reporting Summary [file 41467_2020_18761_MOESM3_ESM.pdf]

nature research

Corresponding author(s): Paul J. Lehner, Yorgo Modis

Last updated by author(s): Aug 28, 2020

Reporting Summary

Nature Research wishes to improve the reproducibility of the work that we publish. This form provides structure for consistency and transparency in reporting. For further information on Nature Research policies, see Authors & Referees and the Editorial Policy Checklist .

Statistics

For all statistical analyses, confirm that the following items are present in the figure legend, table legend, main text, or Methods section.

n/a	Confirmed	
☐	☒	The exact sample size (*n*) for each experimental group/condition, given as a discrete number and unit of measurement
☐	☒	A statement on whether measurements were taken from distinct samples or whether the same sample was measured repeatedly
☐	☒	The statistical test(s) used AND whether they are one- or two-sided<br>*Only common tests should be described solely by name; describe more complex techniques in the Methods section.*
☒	☐	A description of all covariates tested
☐	☒	A description of any assumptions or corrections, such as tests of normality and adjustment for multiple comparisons
☐	☒	A full description of the statistical parameters including central tendency (e.g. means) or other basic estimates (e.g. regression coefficient) AND variation (e.g. standard deviation) or associated estimates of uncertainty (e.g. confidence intervals)
☒	☐	For null hypothesis testing, the test statistic (e.g. *F*, *t*, *r*) with confidence intervals, effect sizes, degrees of freedom and *P* value noted<br>*Give P values as exact values whenever suitable.*
☒	☐	For Bayesian analysis, information on the choice of priors and Markov chain Monte Carlo settings
☒	☐	For hierarchical and complex designs, identification of the appropriate level for tests and full reporting of outcomes
☒	☐	Estimates of effect sizes (e.g. Cohen's *d*, Pearson's *r*), indicating how they were calculated

Our web collection on statistics for biologists contains articles on many of the points above.

Software and code

Policy information about availability of computer code

Data collection	Flow cytometry: BD CellQuest Pro or FACSDIVA; NMR: Bruker Topspin v3.1; X-ray crystallography: autoPROC v4.0, XIA2 v0.4.0.0
Data analysis	Genome profiling (ChIP-seq, CUT&RUN, CUT&Tag): Bowtie2 v2.3.4.3, samtools v1.9, bedtools genomecov v2.27.1 (-bg -scale), SEACR v1.1 (stringent, norm options), RUVseq v1.20, edgeR v3.28.1, UCSC Genome Browser (https://genome.ucsc.edu), IGV v2.4.9, deepTools v3.3.0, Picard (http://broadinstitute.github.io/picard/), HiSat2 v2.1.0, subread v1.6.4; Mass spectrometry: Proteome Discoverer v2.2 (Mascot); NMR: Sparky v3.115, MARS; X-ray crystallography: PHENIX v1.16, COOT v0.8.9.2; Flow cytometry: FlowJo v10

For manuscripts utilizing custom algorithms or software that are central to the research but not yet described in published literature, software must be made available to editors/reviewers. We strongly encourage code deposition in a community repository (e.g. GitHub). See the Nature Research guidelines for submitting code & software for further information.

Data

Policy information about availability of data

All manuscripts must include a data availability statement. This statement should provide the following information, where applicable:

- Accession codes, unique identifiers, or web links for publicly available datasets
- A list of figures that have associated raw data
- A description of any restrictions on data availability

The NMR data were deposited in the Biological Magnetic Resonance Bank, dataset ID 50094. X-ray crystallography data (atomic coordinates and structure factors) were deposited in the Protein Data Bank with code PDB: 6TL1. The original experimental X-ray diffraction images were deposited in the SBGrid Data Bank (data.SBGrid.org), with Data ID 742, DOI:10.15785/SBGRID/742. The CUT&RUN, ChIP and CUT&Tag data will be deposited in the Gene Expression Omnibus (GEO) repository prior to publication.

Field-specific reporting

Please select the one below that is the best fit for your research. If you are not sure, read the appropriate sections before making your selection.

- [x] Life sciences
- [ ] Behavioural & social sciences
- [ ] Ecological, evolutionary & environmental sciences

For a reference copy of the document with all sections, see nature.com/documents/nr-reporting-summary-flat.pdf

Life sciences study design

All studies must disclose on these points even when the disclosure is negative.

Sample size	No sample-size calculations were performed.
Data exclusions	No data was excluded from the analyses in this study.
Replication	All attempts at replication were successful and results from all replicates were self-consistent.
Randomization	There were no human or animal participants in this study. Random allocation did not apply because samples were not subjected to co- or multivariate analysis.
Blinding	There were no human or animal participants in this study. The investigators were not blinded to sample allocation because samples were all analyzed using the same quantitative measurements.

Reporting for specific materials, systems and methods

We require information from authors about some types of materials, experimental systems and methods used in many studies. Here, indicate whether each material, system or method listed is relevant to your study. If you are not sure if a list item applies to your research, read the appropriate section before selecting a response.

Materials & experimental systems

n/a	Involved in the study	
[ ]	[x]	Antibodies
[ ]	[x]	Eukaryotic cell lines
[x]	[ ]	Palaeontology
[x]	[ ]	Animals and other organisms
[x]	[ ]	Human research participants
[x]	[ ]	Clinical data

Methods

n/a	Involved in the study	
[ ]	[x]	ChIP-seq
[ ]	[x]	Flow cytometry
[x]	[ ]	MRI-based neuroimaging

Antibodies

Antibodies used	Wherever possible we provide Resource Identification Portal codes (RRIDs) for antibodies used in this study. The following primary antibodies were used: rabbit α-TASOR (Atlas HPA006735, RRID:AB_1852384 for ChIP, CUT&Tag and Western blot); rabbit α-TASOR (abcam ab224393, for microscopy); rat α-mCherry (Thermo Fisher Scientific, M11217, RRID:AB_2536611); rabbit α-MPP8 (Proteintech, 16796-1-AP, RRID:AB_2266644); rabbit- α-V5 (Abcam, ab27671, RRID:AB_471093); mouse α-Myc (Abcam, ab32, RRID:AB_303599); mouse α-FLAG (Millipore Sigma, F1804, RRID:AB_262044; rabbit α-PPHLN1 (Abcam, ab69569, RRID:AB_1269877); rabbit α-H3K9me3 (abcam ab8898, RRID:AB_306848, for CUT&RUN and CUT&Tag); rabbit α-H3K27me3 (CST C36B11, RRID:AB_2616029, for CUT&RUN positive control); guinea pig α-rabbit IgG (CSB-PA00150E1Gp, for CUT&Run and CUT&Tag); rabbit IgG (CST 2729, RRID:AB_1031062 for ChIP negative control); rabbit α-ORF1p (CST D3W9O, RRID:AB_2800129); mouse α-β-actin (abcam ab8226, RRID:AB_306371).
Validation	Dilutions were made according to manufacturer recommendations unless otherwise stated.

Eukaryotic cell lines

Policy information about cell lines

Cell line source(s)	HeLa and HEK293T cells were obtained from ECACC
Authentication	None of the cell lines were authenticated.
Mycoplasma contamination	Cell lines were routinely tested for mycoplasma contamination using the MycoAlert detection kit (Lonza). All tests were negative.

Commonly misidentified lines (See ICLAC register)	No commonly misidentified cell lines were used.

ChIP-seq

Data deposition

- [x] Confirm that both raw and final processed data have been deposited in a public database such as GEO.
- [x] Confirm that you have deposited or provided access to graph files (e.g. BED files) for the called peaks.

Data access links *May remain private before publication.*	https://zenodo.org/record/3751765<br>All genome profiling data have now been deposited in the Gene Expression Omnibus (GEO) repository under accession codes GSE155693 and GSE95480.
Files in database submission	Bigwig files of rep1 for CUT&RUN H3K9me3 profiling, ChIP and CUT&Tag experiments on TASOR occupancy.
Genome browser session (e.g. UCSC)	Bigwigs available to download for browsing in genome browser of choice.

Methodology

Replicates	Biological replicates as follows: CUT&RUN H3K9me3 - control cells (n=4), TASOR knockout cells (n=4), TASOR KO with WT TASOR complementation (n=2), TASOR KO with Y305A TASOR complementation (n=2); CUT&RUN IgG control (n=4); CUT&Tag TASOR - control cells (n=2), TASOR KO cells (n=2); CUT&Tag IgG control (n=1); CUT&Tag H3K9me3 control (n=1); ChIP TASOR (n=1); ChIP IgG control (n=1); ChIP input (n=1);
Sequencing depth	Mapped read numbers (hg38)<br>CUT&RUN (PE 2x150):<br>H3K9me3 control rep1 - 9.9M; H3K9me3 control rep2 - 9.4M; H3K9me3 control rep3 - 15.9M; H3K9me3 control rep4 - 11.0M; H3K9me3 TASOR KO rep1 - 12.1M; H3K9me3 TASOR KO rep2 - 11.2M; H3K9me3 TASOR KO rep3 - 16.2 M; H3K9me3 TASOR KO rep4 - 11.9M; H3K9me3 WT TASOR comp. rep1 - 12.0M; H3K9me3 WT TASOR comp. rep2 - 10.7M; H3K9me3 Y305A TASOR comp rep1 - 9.5M; H3K9me3 Y305A TASOR comp rep2 - 10.1M; IgG rep1 - 10.5M; IgG rep2 - 31.8M ; IgG rep3 - 9.8M; IgG rep4 - 11.4M<br>CUT&Tag (PE 2x150) [after duplicate removal]:<br>CUT&Tag TASOR - control cells rep1 - 1.7M; CUT&Tag TASOR - control cells rep2 - 2.2M; CUT&Tag TASOR - TASOR KO cells rep1 - 0.7M; CUT&Tag TASOR -TASOR KO cells rep2 - 0.7M; CUT&Tag IgG control - 0.6M<br>ChIP (SE 1x50):<br>ChIP TASOR - 113.1M; ChIP IgG - 57.3M; Input - IgG and TASOR - 59.1M
Antibodies	The following antibodies were used: rabbit α-TASOR (Atlas HPA006735, RRID:AB_1852384 for ChIP, CUT&Tag); rabbit α-H3K9me3 (abcam ab8898, RRID:AB_306848, for CUT&RUN and CUT&Tag); rabbit α-H3K27me3 (CST C36B11, RRID:AB_2616029, for CUT&RUN positive control); guinea pig α-rabbit IgG (CSB-PA00150E1Gp, for CUT&Run and CUT&Tag); rabbit IgG (CST 2729, RRID:AB_1031062 for ChIP negative control).
Peak calling parameters	>>>>bash SEACR.sh H3K9me3-experiment.bedgraph IgG-control.bedgraph norm stringent output_prefix<br><br>SEACR downloaded from https://github.com/FredHutch/SEACR
Data quality	Empirical FDR from SEACR peak calling was ~5%. Only peaks that appeared in more than 1 dataset were included in the differential analysis.
Software	Bowtie2 v2.3.4.3, samtools v1.9, bedtools v2.27.1, SEACR v1.1, RUVseq v1.20, edgeR v3.28.1, UCSC Genome Browser (https://genome.ucsc.edu), IGV v2.4.9, deepTools v3.3.0, Picard (http://broadinstitute.github.io/picard/), HiSat2 v2.1.0, subread v1.6.4.

Flow Cytometry

Plots

Confirm that:

- [x] The axis labels state the marker and fluorochrome used (e.g. CD4-FITC).
- [x] The axis scales are clearly visible. Include numbers along axes only for bottom left plot of group (a 'group' is an analysis of identical markers).
- [ ] All plots are contour plots with outliers or pseudocolor plots.
- [ ] A numerical value for number of cells or percentage (with statistics) is provided.

Methodology

Sample preparation	Cultured cells were fixed in 1% paraformaldehyde (PFA).

Instrument	FACSCalibur or FACSFortessa instruments (BD Bioscience) were used for data collection
Software	BD CellQuest Pro or FACSDIVA was used to collect the data. FlowJo10 was used to analyze the data.
Cell population abundance	N/A
Gating strategy	Live cells were gated on based on FSC and SSC as shown in the attached figure exemplifying the gating strategy; no further gating was performed.

☒ Tick this box to confirm that a figure exemplifying the gating strategy is provided in the Supplementary Information.
