# Supplementary material for: TASOR is a pseudo-PARP that directs HUSH complex assembly and epigenetic transposon control
Source: Nat Commun. 2020 Oct 2;11:4940. doi: 10.1038/s41467-020-18761-6 (PMC7532188; doi:10.1038/s41467-020-18761-6)
Supplement: Supplementary file 4 — Description of Additional Supplementary Files [file 41467_2020_18761_MOESM4_ESM.pdf]

Description of Additional Supplementary Files

File name: Supplementary Data 1

Description: List of TASOR BioID hits with highlights
