# Supplementary material for: TASOR is a pseudo-PARP that directs HUSH complex assembly and epigenetic transposon control
Source: Nat Commun. 2020 Oct 2;11:4940. doi: 10.1038/s41467-020-18761-6 (PMC7532188; doi:10.1038/s41467-020-18761-6)
Supplement: Supplementary file 2 — Peer Review [file 41467_2020_18761_MOESM2_ESM.pdf]

Reviewer #1 (Remarks to the Author):

In the manuscript “TASOR is a pseudo-PARP that directs HUSH complex assembly and epigenetic transposon control” the authors present several lines of evidence supporting a role of TASOR in the structural and transcription control functions of the HUSH complex. They show that TASOR is associated with and helps regulate L1P elements and repetitive exon, through a process involving H3K9me3. They present evidence that the TASOR is a central member fo the HUSH complex and is required for proper assembly. This role is further dissected to reveal the domains involved in some of the key interactions of complex assembly. They identify yeast homologues, which they use as further clues to TASOR function. They also dissect the necessity of MPP8 domains in the transcription repression function of HUSH. Bioinformatic analyses implicate TASOR residues 106-332 as a PARP domain and it exhibits structural homology based on novel structural data but argues that it is likely catalytically inactive. They test this empirically and find no PARP activity under the conditions tested. They demonstrate weak RNA binding of TASOR, which has been previously associated with RNA binding and RNA binding complexes. Finally, they identify a critical loop within the Pseudo-PARP domain. Even a single point mutation within this region can disrupt the function as a repressor.

Overall, the manuscript is accurately presented and makes significant progress in understanding the function and structure of TASOR and the HUSH complex. The manuscript is well written, easy to follow, and grammatically near flawless. The methods are appropriately applied and the resulting data is properly interpreted. There are only a few points that should be addressed prior to publication. The most notable is an extensive lack of clarity about the structural and mechanistic explanation of SETDB1’s role in the complex. This needs to be explained clearly and carefully. I do not find it necessary to have an answer to the remaining questions around SETDB1. The functional observations of the core HUSH complex are sufficient for publication. However, be clear about what evidence supports potential mechanisms by which HUSH induces SETDB1 activity, either directly or indirectly. State what is not known and what remains unresolved by your work. Discuss future work to resolve these issues.

Line 113
It is inaccurate to state that “TASOR binds […] H3K9me3”. It does not bind directly according to your data and later discussion. MPP8 performs this function. This is likely only a matter of verbiage. TASOR indirectly binds H3K9me3 through MPP8, its close binding partner in the HUSH complex. At this point in the manuscript, given the story arc you may want to simply state that TASOR is found to be associated with H3K9me3.

Line 124 (general area thereof)
It seems highly likely that there is more to the story of the different types of genomic localization observed. Although each of these may contain H3K9me3, there is indubitably other distinguishing characteristics that not only define these loci differently but affect the HUSH complex association and possibly function. Did you attempt to localize other histone PTMs to these same loci? For example, H3K14ac is certainly more abundant in the transcriptionally active regions bound by HUSH. How does K9me3K14ac differ from K9me3K14unmodified in MPP8 association? A full exploration down this line of inquiry is outside the scope of the manuscript; however, the current description excludes the almost certain fact that not all H3K9me3 loci are the same.

Figure 1C
Why is there no SetDB1 in this BioID data set? In general, the case for SetDB1 recruitment is a little weak. Also, no ATF7IP is found in the BioID data. It is fine that you have no evidence of direct mechanistic interaction in this data; however, you should declare this as such and make some conjecture as to why. “Notably absent are… This may be due to…”. In general the role of SETDB1 in this system is poorly defined. That is fine. You should make a statement like “The mechanism by which SETDB1 is recruited is not well defined and a direct interaction between HUSH and SETDB1 is not supported by our data; however, our data also suggests a strong functional association.”

MPP8 chromodomain: This is both the at least hypothetical means for the binding for ATF7IP, which in turn recruits SETDB1 to catalyze H3K9me3 (line 84) and is "not strictly essential for HUSH-mediated repression" (line 239). These statements should be discussed and reconciled. Does this suggest an alternative or compensatory mechanism? At least this should be clearly stated as an unresolved question.

Figure 3E: Selective marking of residue #'s, particularly of the binding regions would be nice.

Line 442 and region: This data suggests that this specific region is somehow essential to SETDB1 recruitment. This is quite contradictory to the suggestions above (e.g. MPP8-meATF7IP-SETDB1). These should be presented as contradictory unless you can somehow resolve them.

Line 500: "In light of our NMR data we speculate that the Y305A mutation inhibits a conformational change within the chromatin-engaged HUSH complex, leading to a loss of repression and H3K9me3 deposition." The argument for the mechanism of HUSH repression function goes entirely through H3K9me3. Shouldn't this be "leading to a loss of H3K9me3 deposition and thereby HUSH-mediated repression." Also, does it cause loss of HUSH-chromatin binding or loss of SETDB1 activity from HUSH? At least be clear about the possibilities as to how this *might* occur.

Summary thoughts: Under a MPP8-meATF7IP-SETDB1 mechanism I do not see how to resolve the apparent loss of SETDB1 activity while maintaining H3K9me3 binding. The Y305A mutation would need to cause loss of MPP8 from the complex's which would necessitate the loss of H3K9me3 binding as well. The more likely conclusion is that a MPP8-meATF7IP-SETDB1 mechanism is contradicted by your work. Can you support this further by showing more clearly independence of H3K9me3 binding and H3K9me3 writing? Does TASOR Y305A remain chromatin-bound during the loss of SETDB1 activity?

Line 487: (minor) A citation seems appropriate here.

Line 718 (minor) per standard usage as a unitless figure "m/z" should be set in italics.

I hope my comments improve your manuscript

Best,

Nicolas L. Young
Verna & Marrs McLean Department of Biochemistry & Molecular Biology
Baylor College of Medicine
Houston, TX

Reviewer #2 (Remarks to the Author):

Summary:
In this manuscript, Douse et al. describe results from a number of experiments designed to clarify the composition, structure, and function of the HUSH complex and its relation to H3K9 trimethylation in the human HeLa cell line. Based on biochemical, genetic, and genomic approaches, the authors conclude that HUSH is assembled around the central TASOR subunit, functions as a pseudo-poly-ADP ribose polymerase, and regulates H3K9me3 deposition at repetitive genomic elements (LINE-1 and repetitive exons in transcribed genes).
I believe that the authors have presented thorough and compelling data, and I believe that the

manuscript is well-suited for Nature Communications. However, I have the following concerns:

Major Revisions:
1. More can be learned from the genomic studies presented (CUT&RUN/Tag and RNA-seq) through some additional analyses. In Figure 1B, for example, determining the TASOR-regulated and H3K9me3-marked gene classes (e.g. GO-term analysis), and whether or not the TASOR KO changes expression of genes highlighted. Either RNA-seq (if available/already performed) or site-specific RT-qPCR could answer this question at a couple of loci.
2. In the reporting summary, the authors indicate multiple replicates were performed of all CUT&RUN and CUT&Tag experiments. However, genome-wide data representations (such as heatmaps) are either averaged data or one replicate. This should be made clear, and regardless of which method was chosen to represent these data, the replicates (and correlation of data) should be shown in the supplement so readers can assess the quality of replicates.
3. In Figure 3D, the 1-636 and 1-1000 mutants appear to be much more lowly expressed in the input. While this does not affect the MPP8 IP, but perhaps this could explain the lack of recovery for PPHLN1? Quantitation of replicates to assess expression changes could make for a more compelling argument for rescue experiments without needing to perform additional experiments.

Minor Revisions:
1. In Figure 1A, it is unclear whether the CUT&RUN and IgG experiments come from the knockout or wildtype cell lines. This should be clearly listed.
2. In Figure 1B, I assume this is log10 RPKM, but would like this explicitly stated.
3. The authors identify four factors that interact with TASOR with more peptides relative to HUSH subunits. While the authors discussed NP220 and RPRD2 in the text, why are Ahnak and Tox4 enriched for TASOR interaction?
4. For Supplemental Figure 1B, are percentages made relative to IgG or knockout?
5. Showing heatmaps of CUT&RUN/Tag data over H3K9me3, LINE1, H3K9me3-marked genes, genes that lose H3K9me3 upon TASOR KO, etc in the main text. would present a more compelling case, or complement individual browser tracks in the main text.
6. Is there a functional change associated with loss of TASOR at H3K9me3-regulated genes shown in Figure 1B? Perhaps a change in accessibility or gene expression resulting from the loss of TASOR?
7. Supplemental Figure 1C would be augmented by showing H3K9me3 sorted the same way over these same peaks to determine whether there is a relationship between H3K9me3 level and TASOR occupancy. Additionally, it is unclear to us whether these data are sorted by TASOR ChIP-seq occupancy, CUT&Tag occupancy, or H3K9me3 level.
8. In Supplemental Figure 1D, I question why data were normalized by subtracting the knockout data from the control data, rather than making relative to the IgG control.
9. In Figure 2A, is the greater shift for +1-1085 significant? Is there a better rescue with a shorter peptide? Have the authors performed quantitation or multiple replicates to confirm that this shift is consistent? In panel B, is perhaps MPP8 500-860 only a partial rescue relative to full length?
10. Figure 2B is missing a label for the red trace.
11. As noted above, some mutants in Figure 3D (1-636 and 1-1000) appear to have only modest expression in the input. This expression should either be corrected or commented on.
12. In Figure 5B, there is some variability in these assays. Perhaps Y217A was only moderately functional compared with L184H, which could have been more functional? Showing multiple replicates or quantification of replicates could help explain these findings. If replicates do not alter these findings, is there an explanation the authors could include?
13. For Figure 6D, what the expression of these rescues. Perhaps rescue of WT full length is lowly expressed? It is surprising to see only a partial rescue in KO + WT. Quantitation shows convincingly that ΔPARP and Y305A do not rescue equally or better than the TASOR KO, but the data shown may not be fully reflective of this.
14. In Figure 6E, why is only one replicate is shown for the control and TASOR KO? An IgG control should be performed from both samples, as there could be cell to cell variation. In addition, again normalization should be done relative to IgG, not by subtracting the KO data from that of the sample.

Alternatively, as these data should just be showing H3K9me3 occupancy at these loci, there should be no normalization to data from a different experiment.
15. It is unclear to us why H3K9me3 is bracketed in Figure 6E.
16. Is the signal in the lower half of the heatmap of Figure 6E enrichment for H3K9me3 or merely background signal? Comparing directly with other replicates and/or the IgG control would help to clarify this issue.
17. Although replicates are indicated in Figure 6E, either data are averaged or only one replicate is shown. If only one replicate is shown, other replicates must be shown in the supplement and/or correlation should be shown. If averaged data are shown, individual data should be shown in the supplement.
18. In Figure 6F, the control IgG should be shown on the same scale as the other tracks.

Reviewer #3 (Remarks to the Author):

This is an impressive manuscript investigating some of the unanswered questions regarding the structure and function of the HUSH complex. For example, how is HUSH recruited to euchromatin to set up repressive chromatin marks?

The authors performed CUT&RUN for H3K9me3 marks and identified 393 TASOR-regulated loci, the majority of which corresponded to young, primate-specific L1s. TASOR CUT&RUN did not work but TASOR CUT&TAG peaks were co-occupied by H3K9me3, suggesting that TASOR was recruited to these chromatin sites via MPP8. TASOR binding and H3K9me3 deposition over L1s or other repetitive exons that were targeted by TASOR, correlated with transcription at those sites. To identify proteins localized with TASOR in vivo, the authors did proximity-dependent labeling using BirA-tagged TASOR in TASOR knockout (KO). Hits included RNA processing machinery, consistent with the observation that HUSH regulates transcriptionally active sites and with reports that TASOR is an mRNA-interacting protein.

The authors noted the structural similarity of RITS (Chp1-Tas3) to that of HUSH (Fig. 1D). They then assessed a panel of Tasor deletion mutants for the ability to rescue silencing in Tasor KO cells. Deletion mutants lacking the DUF3715, SPOC, or DomI domains were non-functional. A variant lacking the DomII/PIN domains (deletion of residues 1233-1670) complemented the knockout, indicating that this C-terminal region was not required for transgene repression under the conditions tested. Similar functional mapping was performed with MMP8 deletion mutants. Using triple KO cells the authors showed that the central portion of TASOR is required as an assembly scaffold for the other HUSH components.

The N-terminal DUF3715 domain of Tasor was shown to be required for repression activity, independently of Tasor’s role in assembly of the HUSH complex. Bioinformatic assessment of the DUF3715 domain suggested it resembled a poly ADP-ribose polymerase (PARP) catalytic domain, and solution of its structure by NMR-guided crystallography demonstrated that it is indeed a PARP, though a PARP that lacks activity.

This manuscript provides important new information regarding the structure and function of the HUSH complex. The data is convincing. The manuscript is well written and very interesting to read.

REVIEWER COMMENTS

We thank the two reviewers for their attentive reading of our manuscript and for their positive and constructive comments. We have carefully revised our manuscript and added new display items to address the reviewers' concerns. We have added two supplementary figures (Figs. S6 and S7), along with several new main figure panels (1B, 1C, 6A, 6C). We have also revised the text and figures throughout to address each critique. A copy of the manuscript with all substantive changes to the text highlighted has been attached as part of the review materials. Detailed point-by-point responses to the reviewers' comments follows below.

Reviewer #1 (Remarks to the Author):

In the manuscript "TASOR is a pseudo-PARP that directs HUSH complex assembly and epigenetic transposon control" the authors present several lines of evidence supporting a role of TASOR in the structural and transcription control functions of the HUSH complex. They show that TASOR is associated with and helps regulate L1P elements and repetitive exon, through a process involving H3K9me3. They present evidence that the TASOR is a central member of the HUSH complex and is required for proper assembly. This role is further dissected to reveal the domains involved in some of the key interactions of complex assembly. They identify yeast homologues, which they use as further clues to TASOR function. They also dissect the necessity of MPP8 domains in the transcription repression function of HUSH. Bioinformatic analyses implicate TASOR residues 106-332 as a PARP domain and it exhibits structural homology based on novel structural data but argues that it is likely catalytically
inactive. They test this empirically and find no PARP activity under the conditions tested. They demonstrate weak RNA binding of TASOR, which has been previously associated with RNA binding and RNA binding complexes. Finally, they identify a critical loop within the Pseudo-PARP domain. Even a single point mutation within this region can disrupt the function as a repressor.

Overall, the manuscript is accurately presented and makes significant progress in understanding the function and structure of TASOR and the HUSH complex. The manuscript is well written, easy to follow, and grammatically near flawless. The methods are appropriately applied and the resulting data is properly interpreted. There are only a few points that should be addressed prior to publication. The most notable is an extensive lack of clarity about the structural and mechanistic explanation of SETDB1's role in the complex. This needs to be explained clearly and carefully. I do not find it necessary to have an answer to the remaining questions around SETDB1. The functional observations of the core HUSH complex are sufficient for publication. However, be clear about what evidence supports potential mechanisms by which HUSH induces SETDB1 activity, either directly or indirectly. State what is not known and what remains unresolved by your work. Discuss future work to resolve these issues.

Some key aspects of how SETDB1 functions as a HUSH effector remain unclear and this was reflected in the original manuscript. We agree, however, that it a clarification of the potential mechanisms by which HUSH induces SETDB1 activity and what evidence supports them is warranted. To address this, we have added several lines in the Introduction and Discussion to state more explicitly the possible mechanisms of SETDB1 activation by HUSH, citing the evidence supporting them. We also clearly articulate three specific questions that remain unresolved regarding SETDB1 activation by HUSH. These new sections read as follows:

Introduction (lines 90-94):

"In the current model of HUSH-mediated repression, HUSH regulates both reading and writing of H3K9me3[14]. The MPP8 chromodomain binds K9-trimethylated H3 tail peptide[21] and H3-like mimic sequences found in other proteins including ATF7IP, the nuclear chaperone of SETDB1[22]. It follows that MPP8 binding to methylated ATF7IP recruits SETDB1 to spread H3K9me3 over HUSH targets[23]. However, a read-write mechanism for H3K9me3 spreading involving MPP8 and ATF7IP/SETDB1 must be too simplistic, as TASOR and Periphilin are both essential for HUSH-dependent lentiviral reporter repression, whereas the MPP8 chromodomain is required for establishment but not maintenance of repression[11]. Furthermore and in contrast to classical heterochromatin regulators, HUSH targets are enriched within transcriptionally-active chromatin[15,16]. Hence, key unanswered questions are: how TASOR and Periphilin contribute to HUSH targeting and repression; how H3K9 methylation by SETDB1 is regulated; and whether there are distinct mechanisms to recruit SETDB1 methyltransferase activity to HUSH loci at different stages of development."

Discussion (lines 496-504):
"It remains unclear how recognition of an RNA intermediate by HUSH and H3K9 methylation of HUSH loci by SETDB1/ATF7IP might be linked. The H3K9me3 mark is not solely spread via a simple read-write mechanism involving MPP8 and SETDB1, as the MPP8 chromodomain is not required to maintain pre-established repression but TASOR and Periphilin are[11]. Another domain, such as the TASOR pseudo-PARP, could be required to activate SETDB1. Alternatively, a specific structure or compact physical state of the chromatin, generated by HUSH effector MORC2 [13-15], may be necessary to license target loci for SETDB1 methylation."

Additionally, we have added brief discussion and a new supporting reference in the Results (lines 141-146) citing the potential role histone modifications other than H3K9me3 in HUSH-dependent activation of SETDB1 activity (see "Line 124" response below).

Line 113
It is inaccurate to state that "TASOR binds […] H3K9me3". It does not bind directly according to your data and later discussion. MPP8 performs this function. This is likely only a matter of verbiage. TASOR indirectly binds H3K9me3 through MPP8, its close binding partner in the HUSH complex. At this point in the manuscript, given the story arc you may want to simply state that TASOR is found to be associated with H3K9me3.

We have changed the subheading to "TASOR associates with and regulates H3K9me3 over L1P elements and repetitive exons" (Line 116). We also replaced "H3K9me3-independent binding" with "H3K9me3-independent association" in Line 137.

Line 124 (general area thereof)
It seems highly likely that there is more to the story of the different types of genomic localization observed. Although each of these may contain H3K9me3, there is indubitably other distinguishing characteristics that not only define these loci differently but affect the HUSH complex association and possibly function. Did you attempt to localize other histone PTMs to these same loci? For example, H3K14ac is certainly more abundant in the transcriptionally active regions bound by HUSH. How does K9me3K14ac differ from K9me3K14unmodified in MPP8 association? A full exploration down this line of inquiry is outside the scope of the manuscript; however, the current description excludes the almost certain fact that not all H3K9me3 loci are the same.

We agree that not all H3K9me3 loci will be the same and that the epigenetic context is likely to determine the precise outcome in terms of HUSH-dependent repression. We have added a brief discussion citing the potential role of histone modifications other than H3K9me3 in targeting the methyltransferase activity of SETDB1 to HUSH loci (lines 141-146). We

mention acetylation as an example and cite a new supporting reference (Jurkowska et al, PMID 29234025) along with Liu et al 2018 (Ref. 15).

Figure 1C
Why is there no SetDB1 in this BioID data set? In general, the case for SetDB1 recruitment is a little weak. Also, no ATF7IP is found in the BioID data. It is fine that you have no evidence of direct mechanistic interaction in this data; however, you should declare this as such and make some conjecture as to why. “Notably absent are… This may be due to…”. In general the role of SETDB1 in this system is poorly defined. That is fine. You should make a statement like “The mechanism by which SETDB1 is recruited is not well defined and a direct interaction between HUSH and SETDB1 is not supported by our data; however, our data also suggests a strong functional association.”

In response to this critique we have added a statement that SETDB1, ATF7IP and also MORC2 are all absent from the BioID interactor list. We include as requested some conjecture as to why this might be. The new text (lines 200-204) reads as follows:
“HUSH effectors MORC2 and the SETDB1/ATF7IP complex were absent from the list, suggesting that these factors interact with HUSH transiently and possibly indirectly (e.g. through chromatin). Also absent were proteins usually associated with transcriptionally-inert heterochromatin, reinforcing the model that HUSH resides at a subset of H3K9me3-marked sites.”

MPP8 chromodomain: This is both the at least hypothetical means for the binding for ATF7IP, which in turn recruits SETDB1 to catalyze H3K9me3 (line 84) and is “not strictly essential for HUSH-mediated repression” (line 239). These statements should be discussed and reconciled. Does this suggest an alternative or compensatory mechanism? At least this should be clearly stated as an unresolved question.

It is indeed surprising that we previously found the MPP8 chromodomain to be dispensable for the maintenance of HUSH function (Ref. 11). However, a mutation in the chromodomain that inhibits H3K9me3 binding delayed re-establishment of reporter repression, suggesting that the chromodomain is in fact required to establish HUSH-dependent repression (as opposed to maintenance of a pre-existing repressed state). We have clarified this point in the Results (lines 257-262). The edited text reads as follows:
“Surprisingly, we previously found the chromodomain to be dispensable for the maintenance of HUSH function, although a mutation that inhibits H3K9me3 binding delayed re-establishment of reporter repression[11]. Extending this analysis of MPP8, we found that the first 499 amino acids could be removed altogether without further impairing HUSH maintenance function. However, deletion of an additional 60 residues or the C-terminal ankyrin repeats did abolish HUSH function”

New text in the Introduction and Discussion relating to how SETDB1 is recruited to H3K9me3-marked loci by HUSH (lines 90-94 and 496-504; see above) provides further clarification on this point.

Figure 3E: Selective marking of residue #’s, particularly of the binding regions would be nice.

Selected residues numbers have been added as requested.

Line 442 and region: This data suggests that this specific region is somehow essential to SETDB1 recruitment. This is quite contradictory to the suggestions above (e.g. MPP8-meATF7IP-SETDB1). These should be presented as contradictory unless you can somehow resolve them.

The abovementioned new text in the Discussion (lines 496-504) help clarify this point. In this passage, we explain that our model for how the H3K9me3 mark is not solely spread via a simple read-write mechanism involving MPP8 and SETDB1 but rather via a more complex mechanism involving another domain, such as the TASOR pseudo-PARP, or a specific structure or physical state of chromatin.

Line 500: “In light of our NMR data we speculate that the Y305A mutation inhibits a conformational change within the chromatin-engaged HUSH complex, leading to a loss of repression and H3K9me3 deposition.” The argument for the mechanism of HUSH repression function goes entirely through H3K9me3. Shouldn’t this be “leading to a loss of H3K9me3 deposition and thereby HUSH-mediated repression.” Also, does it cause loss of HUSH-chromatin binding or loss of SETDB1 activity from HUSH? At least be clear about the possibilities as to how this *might* occur.

Our current model is that Y305A affects SEDTDB1 recruitment or activation rather than interfering with chromatin association of the HUSH complex. It is unclear, however, whether the mutation directly affects SETDB1 activation or whether the effect is indirect (e.g. via changes to chromatin structure). We modified the text and added a sentence to expand on how a conformational change inhibited by Y305A might interfere with SETDB1-dependent deposition of H3K9me3 (while avoiding unfounded speculation). The passage in question now reads:

“In light of our NMR data we speculate that the Y305A mutation inhibits a conformational change within the chromatin-engaged HUSH complex, leading to a loss of H3K9me3 deposition and HUSH-mediated repression. Whether the Y305A mutation inhibits SETDB1 activity directly or indirectly (e.g. via changes to chromatin structure) requires further investigation.” (lines 534-538)

The abovementioned changes to the Discussion (lines 496-504) provide additional clarification on this point.

Summary thoughts: Under a MPP8-meATF7IP-SETDB1 mechanism I do not see how to resolve the apparent loss of SETDB1 activity while maintaining H3K9me3 binding. The Y305A mutation would need to cause loss of MPP8 from the complex’s which would necessitate the loss of H3K9me3 binding as well. The more likely conclusion is that a MPP8-meATF7IP-SETDB1 mechanism is contradicted by your work. Can you support this further by showing more clearly independence of H3K9me3 binding and H3K9me3 writing? Does TASOR Y305A remain chromatin-bound during the loss of SETDB1 activity?

Based on the data we present in Figs. 6C, S5B and S5C we conclude that with the Y305A and deltaPARP TASOR variants, HUSH remains associated with chromatin despite the loss of H3K9me3 writing activity caused by those variants. The changes to the Discussion outlined above (lines 496-504 and lines 534-538) articulate the open question of how H3K9me3 binding by HUSH and H3K9 methylation by SETDB1 are related and suggest possible mechanisms. However, how HUSH is recruited to chromatin remains a key open question that we could not fully resolve in this study.

Line 487: (minor) A citation seems appropriate here.

We have added reference (Ref. 12).

Line 718 (minor) per standard usage as a unitless figure “m/z” should be set in italics.

As requested, m/z has been set in italics.

I hope my comments improve your manuscript

Best,

Nicolas L. Young
Verna & Marrs McLean Department of Biochemistry & Molecular Biology
Baylor College of Medicine
Houston, TX

We are very grateful for your helpful comments. We have added a statement in the acknowledgements thanking Dr. Young and the two anonymous reviewers for their constructive and helpful comments.

Reviewer #2 (Remarks to the Author):

Summary:
In this manuscript, Douse et al. describe results from a number of experiments designed to clarify the composition, structure, and function of the HUSH complex and its relation to H3K9 trimethylation in the human HeLa cell line. Based on biochemical, genetic, and genomic approaches, the authors conclude that HUSH is assembled around the central TASOR subunit, functions as a pseudo-poly-ADP ribose polymerase, and regulates H3K9me3 deposition at repetitive genomic elements (LINE-1 and repetitive exons in transcribed genes).
I believe that the authors have presented thorough and compelling data, and I believe that the manuscript is well-suited for Nature Communications. However, I have the following concerns:

Major Revisions:
1. More can be learned from the genomic studies presented (CUT&RUN/Tag and RNA-seq) through some additional analyses. In Figure 1B, for example, determining the TASOR-regulated and H3K9me3-marked gene classes (e.g. GO-term analysis), and whether or not the TASOR KO changes expression of genes highlighted. Either RNA-seq (if available/already performed) or site-specific RT-qPCR could answer this question at a couple of loci.

In response to this critique we have performed additional analysis on our genomic data and added citations of other studies, which address some of the points raised. We performed GO-term analysis on TASOR-regulation and H3K9me-marked genes, but it only highlighted ZNF genes, which are already discussed in the text. Regarding the effect of TASOR KO on expression of highlighted genes, loss of HUSH-dependent H3K9me3 marks in TASOR KO cells was reported in previous transcriptional profiling studies to cause modest increases in transcript levels over ZNF genes (1.1-1.8-fold increase, PMID 26022416) and over genes harboring intronic L1s or (2-9-fold increase, PMID 29211708). We plan to assess the consequences of H3K9me3 on transcription and transcription dynamics in detail in a separate study. Conventional RNA-seq protocols may not be best suited for this. For example, it would be useful to measure nascent transcript levels (e.g. by mNET-seq) and capture chromatin-associated RNA (e.g. by DRIP-seq). We consider such work to be of great interest to the field but beyond the scope of the current study.

2. In the reporting summary, the authors indicate multiple replicates were performed of all CUT&RUN and CUT&Tag experiments. However, genome-wide data representations (such

as heatmaps) are either averaged data or one replicate. This should be made clear, and regardless of which method was chosen to represent these data, the replicates (and correlation of data) should be shown in the supplement so readers can assess the quality of replicates.

As requested, we have added replicate heatmaps to the main figures. We now show at least two replicates (though four were performed in total for WT/KO anti-H3K9me3) so readers can assess their quality (Fig. 1B in the revised manuscript). We have also added a new supplementary figure, Fig. S6, showing correlation plots comparing the replicates. We state in the Methods (lines 632-636) that "CUT&RUN experiments to assess H3K9me3 regulation were done with four replicates (control and TASOR KO cells, used to define TASOR-regulated peaks) or two replicates (WT and Y305A complemented cells, used to assess H3K9me3 complementation over TASOR-regulated peaks)".

3. In Figure 3D, the 1-636 and 1-1000 mutants appear to be much more lowly expressed in the input. While this does not affect the MPP8 IP, but perhaps this could explain the lack of recovery for PPHLN1? Quantitation of replicates to assess expression changes could make for a more compelling argument for rescue experiments without needing to perform additional experiments.

The expression levels of the TASOR variants are indeed variable, with 1-636 and 1-1000 poorly expressed, deltaDomI highly expressed. These differences in expression level were reproduced in replicate experiments, and are likely attributable to destabilization of the protein fold in certain truncation mutants. They do not, however, correlate with PPHLN1 recovery in the co-IP as TASOR 1-505 and 1-800, which have intermediate expression levels, also fail to pull down PPHLN1. In response to this comment, we have added the following statements in the text (lines 304-307):

"We noted some variability in the abundance of TASOR variants. TASOR(1-636) and TASOR(1-1000) were barely detectable suggesting these variants are unstable. There was no correlation between the relative expression level of the variants and their ability to support HUSH complex assembly."

Minor Revisions:
1. In Figure 1A, it is unclear whether the CUT&RUN and IgG experiments come from the knockout or wildtype cell lines. This should be clearly listed.

We have added labels to Fig. 1A specifying which control tracks are from wildtype cells and which are from knockout cells. The IgG experiments were performed in wildtype cells and knockout cells; heatmaps for both are shown in Fig. 1B.

2. In Figure 1B, I assume this is log10 RPKM, but would like this explicitly stated.

Yes it was log10 RKPM, now stated explicitly in the axis label (Fig. 1D in the revised manuscript).

3. The authors identify four factors that interact with TASOR with more peptides relative to HUSH subunits. While the authors discussed NP220 and RPRD2 in the text, why are Ahnak and Tox4 enriched for TASOR interaction?

Whether AHNAK or TOX4 play a role in HUSH assembly or activity remains unclear. The Uniprot annotation for human AHNAK identifies it as an RNA-binding protein involved in splicing regulation (based on an RNA-protein interactome screen, PMID 22658674, and on PMID 21940993). TOX4 plays an important role in regulating transcription (by dephosphorylating the C-terminal tail of Pol II- see PMID 24204300, 20941529, 24591642).

We have added TOX4 to the list of proteins "associated with regulating mRNA processing or RNA polymerase II activity" in the Results section (line 205), and AHNAK to the list of non-L1 targets with TASOR-dependent H3K9me3 deposition (line 153).

4. For Supplemental Figure 1B, are percentages made relative to IgG or knockout?

The figure (now main Fig. 1C) represents the different types of TEs that overlapped with TASOR loci, which are defined as those peaks which have lost H3K9me3 in a TASOR KO sample relative to control, from four independent experiments. Because TASOR loci extend over many thousands of bases, most TASOR-regulated loci overlap with more than one repeat. Only unique repeats (i.e. unique genomic loci) were taken into consideration. Of all the TE repeats that overlap with TASOR-regulated loci, 86.1% of them are LINE1s, 8.8% of them are LTRs, 2.7% are DNA transposons and 2% are LINE2, with a small proportion of "other" repeats.

5. Showing heatmaps of CUT&RUN/Tag data over H3K9me3, LINE1, H3K9me3-marked genes, genes that lose H3K9me3 upon TASOR KO, etc in the main text. would present a more compelling case, or complement individual browser tracks in the main text.

We now show two replicates of each of these heatmaps in a main figure, Fig. 1B in the revised submission.

6. Is there a functional change associated with loss of TASOR at H3K9me3-regulated genes shown in Figure 1B? Perhaps a change in accessibility or gene expression resulting from the loss of TASOR?

As outlined in the response to Major Revision 1 above, loss of HUSH-dependent H3K9me3 marks in TASOR KO cells was reported previously to cause small increases in expression of ZNF genes (1.1-1.8-fold increase, PMID 26022416) and intronic LINE1 elements (2-9-fold increase, PMID 29211708). We have not examined changes in accessibility – this is an excellent suggestion for future work. We also plan to assess the consequences of H3K9me3 on transcription by RNA-seq in detail in a separate study.

7. Supplemental Figure 1C would be augmented by showing H3K9me3 sorted the same way over these same peaks to determine whether there is a relationship between H3K9me3 level and TASOR occupancy. Additionally, it is unclear to us whether these data are sorted by TASOR ChIP-seq occupancy, CUT&Tag occupancy, or H3K9me3 level.

We have moved this panel up to main Figure 1B in response to Major Revision 2 and Minor Revision 6 above. The heatmaps are now all sorted by H3K9me3 level, which is now clearly stated. We also show two replicates for each experiment.

8. In Supplemental Figure 1D, I question why data were normalized by subtracting the knockout data from the control data, rather than making relative to the IgG control.

Using the anti-TASOR antibody in a TASOR KO line is, we think, the best control for CUT&Tag, although we also performed IgG controls. Analyzing TASOR binding in TASOR-KO cells helps to identify instances of the so-called 'ATAC-seq artifact' in CUT&Tag. This occurs when the pA-Tn5 reagent binds to open chromatin leading to non-specific transpositions, for example over promoters. An example of this is shown Fig. 1A, in the left hand screenshot, where a sharp TASOR peak over the promoter of TMEM87A is present in both Control and KO cells). This is a known limitation of the technique (discussed extensively at https://www.protocols.io/view/bench-top-cut-amp-tag-z6hf9b6). In our experience, and according to developers of the technique, different antibodies can lead to

slightly different ATAC-like behavior. This is probably caused by the relative concentrations of antibody, pA-Tn5, and genomic target sites. Using the TASOR antibody in a TASOR KO cell line is – in our judgement – the best control for this experiment because it accounts for non-specific antibody binding but also ensures the antibody concentration is the same. Using the IgG control for the heatmaps (now shown in Fig. S1B) could be done, but it would make the data look better than it is since the ATAC-like artifact is less prominent in this control.

9. In Figure 2A, is the greater shift for +1-1085 significant? Is there a better rescue with a shorter peptide? Have the authors performed quantitation or multiple replicates to confirm that this shift is consistent? In panel B, is perhaps MPP8 500-860 only a partial rescue relative to full length?

There are indeed slight variations in the extent of rescue for different complementing variants. Although these variations were obtained in replicate experiments, there was no correlation with protein expression levels (see also response to Major Revision 3 above). On balance, identifying MPP8 560-860 as partially rescuing or 1-1085 rescuing to a greater extent that wildtype would in our view be overinterpreting the data at this stage.

10. Figure 2B is missing a label for the red trace.

Thank you for spotting this- we have restored the red label, which had been deleted by mistake.

11. As noted above, some mutants in Figure 3D (1-636 and 1-1000) appear to have only modest expression in the input. This expression should either be corrected or commented on.

As outlined in the response to Major Revision 3, we have added a brief discussion about the variability in express levels of different variants, including the low expression levels of 1-636 and 1-1000 (lines 304-307). The edited text reads as follows:

"We noted some variability in the abundance of TASOR variants. TASOR(1-636) and TASOR(1-1000) were barely detectable suggesting these variants are unstable. There was no correlation between the relative expression level of the variants and their ability to support HUSH complex assembly."

12. In Figure 5B, there is some variability in these assays. Perhaps Y217A was only moderately functional compared with L184H, which could have been more functional? Showing multiple replicates or quantification of replicates could help explain these findings. If replicates do not alter these findings, is there an explanation the authors could include?

This critique is analogous to Minor Revision 9 above. Here too, we acknowledge that there are indeed slight variations in the extent of rescue for the two complementing variants. Although these variations were obtained in replicate experiments, we don't have a clear explanation for why L184H might be more functional that Y217A. Determining crystal structures of these two variants or conducting additional functional assays might provide some clues (but fall beyond the scope of this study in our view). On balance, with the currently available dataset, any explanation for the slightly different profiles of the histograms in Fig. 5B would be excessively speculative.

13. For Figure 6D, what the expression of these rescues. Perhaps rescue of WT full length is lowly expressed? It is surprising to see only a partial rescue in KO + WT. Quantitation shows

convincingly that ∆PARP and Y305A do not rescue equally or better than the TASOR KO, but the data shown may not be fully reflective of this.

Yes, WT rescue and other active-variant rescues tend to be lowly expressed relative to inactive variants. Indeed, we observed higher expression of Y305A and deltaPARP variants relative to WT (based on confocal IF in Fig. 6C, mass spectrometry in Fig. S5C and Western blots). We have reported higher expression of non-functional mutants relative to wildtype previously for HUSH effector MORC2 (Ref. 13). We suspect there is some form of autoregulation of HUSH protein levels when the complex is active, but we do not yet understand the underlying mechanism. We therefore tried to avoid speculation regarding variability of expression levels, as there are potential confounding factors. What is clear is that despite being expressed at higher levels than WT – the PARP deletion variant and point mutant Y305A fail to rescue the functions of HUSH.

14. In Figure 6E, why is only one replicate is shown for the control and TASOR KO? An IgG control should be performed from both samples, as there could be cell to cell variation. In addition, again normalization should be done relative to IgG, not by subtracting the KO data from that of the sample. Alternatively, as these data should just be showing H3K9me3 occupancy at these loci, there should be no normalization to data from a different experiment.

We now show a second replicate for the heatmaps in question (four replicates were performed for these experiments).

As requested, we have added IgG controls for both the Control and TASOR KO samples.

We no longer compare or normalize the rescues with WT and Y305A to KO. Hence, we moved the Control and KO cell data to Fig. 1B in the revised manuscript. Fig 6E now just shows H3K9me3 occupancy at the TASOR-regulated loci for the WT and Y305A rescues.

15. It is unclear to us why H3K9me3 is bracketed in Figure 6E.

In light of the changes to Fig. 6 outlined in the response to the previous point, this no longer applies.

16. Is the signal in the lower half of the heatmap of Figure 6E enrichment for H3K9me3 or merely background signal? Comparing directly with other replicates and/or the IgG control would help to clarify this issue.

A direct comparison of pairs of replicates and the IgG control for this data is now shown in Fig. 1B. The H3K9me3 signal in the KO is clearly distinguishable above the background.

The observation that at HUSH loci there is residual H3K9me3 in the KO is interesting. Though we do not investigate this further here, the KAP1/TRIM28 system may be a source of residual H3K9me3, as suggested in PMID 29728366. Alternatively, a methyltransferase other than SETDB1 could be responsible for deposition of these H3K9me3 marks (e.g. SUV39H1/2).

17. Although replicates are indicated in Figure 6E, either data are averaged or only one replicate is shown. If only one replicate is shown, other replicates must be shown in the supplement and/or correlation should be shown. If averaged data are shown, individual data should be shown in the supplement.

As detailed in the response to Major Revision 2, two replicates are now shown for all heatmaps and correlations between the replicates are shown in a new supplementary figure

(Fig. S6).

18. In Figure 6F, the control IgG should be shown on the same scale as the other tracks.

Fig. 6F now shows the control IgG on the same scale as the other tracks.

Reviewer #3 (Remarks to the Author):

This is an impressive manuscript investigating some of the unanswered questions regarding the structure and function of the HUSH complex. For example, how is HUSH recruited to euchromatin to set up repressive chromatin marks?

The authors performed CUT&RUN for H3K9me3 marks and identified 393 TASOR-regulated loci, the majority of which corresponded to young, primate-specific L1s. TASOR CUT&RUN did not work but TASOR CUT&TAG peaks were co-occupied by H3K9me3, suggesting that TASOR was recruited to these chromatin sites via MPP8. TASOR binding and H3K9me3 deposition over L1s or other repetitive exons that were targeted by TASOR, correlated with transcription at those sites. To identify proteins localized with TASOR in vivo, the authors did proximity-dependent labeling using BirA-tagged TASOR in TASOR knockout (KO). Hits included RNA processing machinery, consistent with the observation that HUSH regulates transcriptionally active sites and with reports that TASOR is an mRNA-interacting protein.

The authors noted the structural similarity of RITS (Chp1-Tas3) to that of HUSH (Fig. 1D). They then assessed a panel of Tasor deletion mutants for the ability to rescue silencing in Tasor KO cells. Deletion mutants lacking the DUF3715, SPOC, or DomI domains were non-functional. A variant lacking the DomII/PIN domains (deletion of residues 1233-1670) complemented the knockout, indicating that this C-terminal region was not required for transgene repression under the conditions tested. Similar functional mapping was performed with MMP8 deletion mutants. Using triple KO cells the authors showed that the central portion of TASOR is required as an assembly scaffold for the other HUSH components.

The N-terminal DUF3715 domain of Tasor was shown to be required for repression activity, independently of Tasor’s role in assembly of the HUSH complex. Bioinformatic assessment of the DUF3715 domain suggested it resembled a poly ADP-ribose polymerase (PARP) catalytic domain, and solution of its structure by NMR-guided crystallography demonstrated that it is indeed a PARP, though a PARP that lacks activity.

This manuscript provides important new information regarding the structure and function of the HUSH complex. The data is convincing. The manuscript is well written and very interesting to read.

We thank the reviewer for their careful reading of the manuscript and for the enthusiastic and supportive comments.

REVIEWERS' COMMENTS:

Reviewer #1 (Remarks to the Author):

The authors have responded appropriately and effectively to the previous reviewer comments. The changes made to the manuscript clarify several important points where gaps in knowledge persist and increased precision of language in describing functional relationships. Overall, the manuscript presents convincing evidence that reveals greater functional and mechanistic details of TASOR and the HUSH complex. The evidence is presented with a high degree of transparency (e.g. with easy access to uncropped images etc.). The manuscript is now suitable for publication.

Nicolas L. Young
Verna & Marrs McLean Department of Biochemistry & Molecular Biology
Baylor College of Medicine

Reviewer #2 (Remarks to the Author):

The authors have thoroughly addressed all my original concerns and I think that this study is suitable for publication in Nature Communications. It is very thorough, well controlled, and has compelling findings.
